# Beneficial effect of voluntary physical exercise in Plakophilin2 transgenic mice

**DOI:** 10.1371/journal.pone.0252649

**Published:** 2021-06-04

**Authors:** Karin P. Hammer, Julian Mustroph, Teresa Stauber, Walter Birchmeier, Stefan Wagner, Lars S. Maier

**Affiliations:** 1 University Hospital Regensburg, Internal Medicine II, Regensburg, Germany; 2 Max-Delbrueck-Centre for Molecular Medicine, Berlin, Germany; Rush University Medical Center, UNITED STATES

## Abstract

Arrhythmogenic right ventricular cardiomyopathy is a hereditary, rare disease with an increased risk for sudden cardiac death. The disease-causing mutations are located within the desmosomal complex and the highest incidence is found in plakophilin2. However, there are other factors playing a role for the disease progression unrelated to the genotype such as inflammation or exercise. Competitive sports have been identified as risk factor, but the type and extend of physical activity as cofactor for arrhythmogenesis remains under debate. We thus studied the effect of light voluntary exercise on cardiac health in a mouse model. Mice with a heterozygous PKP2 loss-of-function mutation were given the option to exercise in a running wheel which was monitored 24 h/d. We analyzed structural and functional development *in vivo* by echocardiography which revealed that neither the genotype nor the exercise caused any significant structural changes. Ejection fraction and fractional shortening were not influenced by the genotype itself, but exercise did cause a drop in both parameters after 8 weeks, which returned to normal after 16 weeks of training. The electrophysiological analysis revealed that the arrhythmogenic potential was slightly higher in heterozygous animals (50% vs 18% in wt littermates) and that an additional stressor (isoprenaline) did not lead to an increase of arrhythmogenic events pre run or after 8 weeks of running but the vulnerability was increased after 16 weeks. Exercise-induced alterations in Ca handling and contractility of isolated myocytes were mostly abolished in heterozygous animals. No fibrofatty replacements or rearrangement of gap junctions could be observed. Taken together we could show that light voluntary exercise can cause a transient aggravation of the mutation-induced phenotype which is abolished after long term exercise indicating a beneficial effect of long term light exercise.

## Introduction

Arrhythmogenic right ventricular cardiomyopathy (ARVC) is a severe cardiac disease, leading to right ventricular dilation with a risk for sudden cardiac death (SCD), ventricular arrhythmias and syncope. Although ARVC is classified as a rare disease, it is moving into the spotlight as it poses a great risk for SCD among young active athletes during exercise. The vast majority of cases can be traced back to mutations of desmosomal proteins, with plakophilin2 (PKP2) having the highest incidence (about, 65% of mutations, [1, 2]). Diagnosis and prognosis is constantly evolving and new studies help to improve the criteria system constantly [3]. In 1994, Task Force criteria were published with a conundrum of different factors to be taken into account such as familial history, structural, and functional data [4]. An update was published in 2010 as the experience has uncovered potential misdiagnosis based on the original criteria [5]. However, the experience of the last years indicates a larger involvement of the left ventricle as opposed to the right ventricle that had been the focus previously. The combination of criteria required for a definite diagnosis reveals the diversity of the disease as well as the difficulty for risk stratification, which often relies on age, sex and the occurrence of previous adverse events such as syncope or non-sustained tachycardia [6]. Factors other than the disease-causing mutations seem to play a crucial role for the disease progression, such as additional mutations (found in 88% of cases [1]), inflammation [7], or exercise [8].

The role of competitive sports is critical for the disease progression, as it is well known that a significant portion of SCD in athletes is caused by ARVC [9, 10]. However, the type and extend of physical exercise that might be most detrimental for the disease progression remains under discussion. Physical strain has been shown to increase right ventricular wall tension, thereby contributing to acute contractile dysfunction [11] and several reports showed correlations between endurance as well as highly dynamic exercise (as opposed to static exercise) and an increased risk for arrhythmia [12–14]. On the other hand, Ruwald and colleagues found that recreational sports, even if highly dynamic, do not increase the arrhythmogenic risk in ARVC [15]. Up to date it remains a difficult task to decide whether recreational sports can be permissive in asymptomatic or lightly symptomatic patients especially for young, active patients. In 2004, the AHA released a statement suggesting only light recreational activity that should exclude progressive elements aiming at improving performance, competitive or highly dynamic activities, and sports that could cause injury to oneself or others [16].

Animal models have been used to study the role of exercise on adverse cardiac remodeling without the additional predisposition of a desmosomal mutation and found that excessive training can induce adverse structural (e.g. collagen deposition, fibrosis and eccentric hypertrophy) and functional (e.g. increased arrhythmogenic potential and diastolic dysfunction) changes in the heart [17, 18]. Different models have studied the effect of physical exercise on the disease progression in animals carrying a desmosomal mutation. One crucial point when comparing the different outcome of these studies is the baseline condition. A homozygous knockout mouse model of the desmosomal protein plakophilin-2 (PKP2) has been shown to be lethal at an early embryonic state [19], while inducible homozygous knockouts show an overt phenotype (i.e. a disrupted cellular Ca handling) already at rest, albeit without structural changes [20]. Heterozygous PKP2 knockouts have either been described as asymptomatic or with alterations in Ca handling [19, 21]. Variations at baseline may influence the cellular effects of physical exercise, e.g. on eNOS signaling, and might thus influence the outcome. In addition, the type of training plays a crucial role when studying the effects of physical activity on the disease progression and arrhythmogenesis in ARVC models. Swimming or forced running are established models to force physical exercise simulating competitive sports but probably also pose an additional stressor for the animals and might thus influence the detected response. Also, the total duration of exercise exposure might play a role for the outcome and most animal studies on the effects of exercise on the disease progression have been of a shorter duration but could nevertheless detect early changes in the right ventricular function [22]. Potentially maladaptive processes might occur only at a later stage and could be masked or compensated for during the first weeks of training.

The aim of this study was to test whether voluntary physical exercise, that most likely does not add another unaccounted stressor and is more comparable to recreational sports, can induce a phenotype in mice with a heterozygous PKP2 knock-out that is not detectable in resting animals. Such data will add to the knowledgebase about the role of exercise on the disease onset and most importantly whether recreational sports are advisable for asymptomatic carriers of desmosomal mutations.

## Material and methods

All experiments were conducted according to institutional and governmental regulations for animal use investigations conformed the directive 2010/63/EU of the European Parliament. The study follows the Guide for the Care and Use of Laboratory Animals published by the US National Institutes of Health (NIH Publication No. 85–23, revised 1985) and to local institutional guidelines. The study was approved by the Government of Unterfranken, Germany.

### Experimental model

For this study mice harboring a heterozygous deletion of plakophilin2 (PKP2^+/-^) in a C57BL/6J background [19] were bred at the central animal research facility of the University Medical Center Regensburg and selected for the study a minimum age of 20 weeks. The animals were housed at a 12-hour light-dark cycle and standard laboratory chow and water were provided ad libitum. The animals were transferred into a cage with a running wheel attached, which was equipped with a counter to track the running activity of each individual mouse. A subset of animals was kept in standard cages without running wheels as sedentary control. The mice were able to access the running wheel for either 8 or 16 weeks and were then sacrificed for the final experiments.

### Echocardiography

Echocardiography was performed as described before [23] at time point zero before the animals were transferred into the running or sedentary cages and again after 8 and 16 weeks of voluntary activity or resting in the cages.

Briefly, two-dimensional M-mode echocardiography of the left ventricle (LV) was performed using the Vevo 3100 Visualsonics Fujifilm MX400 imaging system (Fujifilm VisualSonics Inc., Toronto, Canada). Mice were anaesthetized with 1.5–2.5% isoflurane and 97.5–98.5% O_2_ via a nose cone. Heart rate was monitored to maintain comparable values between animals of 450±50 bpm under anesthesia. The heart was imaged in the parasternal long axis and short axis view. Digital images were analyzed with Vevo LAB 3.0.0 Software.

### Electrophysiology study

The mice were anesthetized using intraperitoneal injections of medetomidine (0.5 mg/kg), midazolam (5 mg/kg) and fentanyl (0.05 mg/kg body weight). During electrophysiology (EP) studies the mouse body temperature was monitored by an intrarectal probe and controlled using a mousepad circuit board equipped with a heating element (Mousepad, THM 100, Indus Instruments, USA). All studies were performed at 37.0±0.5°C. We used a Millar 1.1F octapolar EP catheter (EPR-800; Millar Instruments) inserted via the right jugular vein, as previously described [24]. A computer-based data acquisition system (Powerlab 16/35; ADI instruments) was used to record a 1-lead body surface ECG and 4 intracardiac bipolar electrograms (Labchart Pro software, version 7; AD Instruments). Right ventricular pacing was performed using 2 ms current pulses (400 mA) delivered by an external stimulator (STG-3008FA; Multi Channel Systems). Inducibility of ventricular arrhythmias was tested by decremental burst pacing. Burst pacing started at a 40 ms cycle length, decreasing by 2 ms every 2 seconds to a cycle length of 20 ms. Burst pacing was repeated one minute after the previous burst concluded or the termination of arrhythmias. Burst pacing was performed for a total of three times in each mouse. Ventricular arrhythmias were defined as the occurrence of rapid ventricular potentials that occurred independent from atrial potentials and displayed altered QRS morphology. Ventricular arrhythmias were considered significant if they at least occurred as triplets. Burst stimulation was repeated 3 min after intraperitoneal injection of isoproterenol (2 mg/kg body weight). At the end of the experiment, mice were sacrificed by cervical dislocation, while still under general anesthesia.

### Tissue collection and cell isolation

After the electrophysiological analysis the animals were sacrificed while still under anesthesia and the hearts quickly excised and transversely divided into three parts to be used for histological and protein expression analysis, where the atria had been removed and discarded. One part was snap frozen in liquid nitrogen and stored at -80°C until the tissue was used for Western Blot analysis, another part was embedded in glycerin-based media (Tissue-Tek, Sakura Finetek Germany GmbH, Staufen, Germany) and frozen in liquid nitrogen and stored at –80°C until cryosectioning was performed. The third section was transferred into paraformaldehyde for paraffin embedding and sectioning.

For cell isolation, mice were anaesthetized with isoflurane, and hearts quickly excised after cervical dislocation. The hearts were mounted on a Langendorff perfusion system and retrogradely perfused with nominally Ca-free solution containing (in mM): 113 NaCl, 4.7 KCl, 0.6 KH_2_PO_4_, 0.6 Na_2_HPO_4_, 1.2 MgSO_4_, 12 NaHCO_3_, 10 KHCO_3_, 10 HEPES, 30 taurine, 10 BDM (2,3 butanedione monoxime), 5.5 glucose, 0.032 phenol-red for 4 min (37°C, pH 7.4). Then, 7.5 mg/ml liberase^™^ (Roche diagnostics, Mannheim, Germany), trypsin 0.6%, and 0.125 mM CaCl_2_ were added and the hearts perfused until they became flaccid. Ventricular tissue was collected in perfusion buffer containing 5% bovine calf serum, cut into small pieces, dispersed and filtered, until no solid tissue was left. After Ca reintroduction by stepwise increasing [Ca] from 0.1 mM to 0.8 mM, cardiomyocytes were plated on laminin coated glass coverslips. Cells were allowed to settle and attach to the coating for 30 min at room temperature (RT) before they were used for the experiments.

### Ca-signaling and cellular contractions

All experiments were performed at RT in normal Tyrode solution (NT) containing (mM): KCl 4, NaCl 140, MgCl_2_ 1, HEPES 5, Glucose 10 and CaCl_2_ 2. Ca measurements were performed in isolated, intact cardiac myocytes loaded with 2 μM Fura2-AM for 15 min on an inverted epifluorescence microscope (IonOptix). Excitation at alternating wavelengths of 340/380 nm resulted in emission that was detected with a photomultiplier at <515 nm for each excitation wavelength. The sarcomere length was recorded with a video camera and calculated by Fourier transformation of the intensity profile from the brightfield image. The cells were field stimulated with platinum bath electrodes at 0.5 Hz to steady state before Ca and contractions were recorded simultaneously. The data are presented as emission ratio of the alternating excitation wavelengths 340/380 and the sarcomere length as nm.

SR Ca load was determined by rapid caffeine application (10 mM) after cells had been paced to steady state at 0.5 Hz.

### Histology

For the analysis of fat infiltration in the cardiac tissue, cryosections were cut, mounted on glass slides and air dried. After washing, the cells were first labelled with oil red to label the lipids and subsequently the nuclei were labelled with hematoxylin. The slides were then recorded with a microscope and the images analyzed using metamorph software to calculate the percentage of oil within the section.

The degree of fibrosis of the tissue was studied in sections from paraffin embedded tissue. The sections were mounted on glass slides and after deparaffination and rehydration stained with Weigert’s solution, Fuchsin and Aniline Blue. Collagen appears blue, while muscle cells appear red. The slides were recorded on a microscope and the image analysis performed with HistoQuest Software to calculate the percentage of collagen in the tissue sections.

A subset of paraffin sections was used for immunohistochemistry to study the distribution of connexin 43 (Cx43) in the ventricular tissue. The sections were labelled with anti-Cx43 (1:400, Alomone Labs, Jerusalem, Israel) and Alexa488 (1:500, Invitrogen) and subsequently analyzed with a confocal microscope. The confocal images were loaded into ImageJ and percentage of labeling at the intercalated disc vs. the cytosol was calculated. The clear accumulation of the connexins at the ID enabled us to calculate the relative fluorescence intensity without counterstain of the membranes and reduce loss of signal by using a long pass filter and exclude any potential bleed through from the second channel.

### Western blot

Cardiac tissue was homogenized in Tris buffer containing (in mM): 20 Tris-HCl, 200 NaCl, 20 NaF, 1 Na3VO4, 1 DTT, 1% Triton X-100 (pH 7.4), complete protease inhibitor cocktail (Roche diagnostics, Mannheim, Germany) and phosphatase-inhibitor mixture (PhosSTOP, Roche). Protein concentration was determined by BCA assay (Sigma-Aldrich Co., St. Louis, MO, USA). Denatured proteins were separated on SDS-polyacrylamide gels (5–12%) and transferred to nitrocellulose membranes (GE Health Care, Chalfont St Giles, UK). Specific proteins were detected using RyR2 (1:10,000, Sigma-Aldrich Co., St. Louis, MO, USA), anti-S2814 (1:3,000), anti-NCX1 (1:500, abcam plc, Cambridge, UK), anti-SERCA2a (1:20,000, ABR Affinity BioReagents, Golden, CO, USA), anti-PLB (1:1,000, Badrilla Ltd., Leeds, UK), anti-PT-17 (1:1,000, Badrilla Ltd., Leeds, UK), anti-PS-16 (1:1,000, Merck KGaA, Darmstadt, Germany) and anti-GAPDH (1:30,000, Biotrend Chemikalien GmbH, Cologne, Germany) antibodies followed by HRP-conjugated donkey anti-rabbit and sheep anti-mouse IgG antibodies (GE Health Care, Chalfont St Giles, UK). Chemiluminescent detection was performed with ImmobilonTM Western Chemiluminescent HRP Substrate (Millipore Corporation, Billerica, MA, USA). Expression levels of the proteins were normalized to GAPDH. Since the total of all analyzed samples could not be analyzed on a single gel, we used reference samples on both gels to normalize the data and compare the average protein expression levels.

### Statistics

Data are presented as mean ± SEM. Before any statistical analysis was performed, the normal distribution of the data analyzed was tested using the Shapiro-Wilk test. Statistical analyses were performed according to the experimental setup, sample size and variability within the groups. For setups where two groups are compared, an unpaired students t-test has been performed, for all other data a mixed effects analysis or a two-way ANOVA with Geisser-Greenhouse correction was performed. The statistical analysis of arrhythmia occurrence during electrophysiological studies was performed by Chi-square test. P<0.05 was considered statistically significant. Analyses and graphs were generated using GraphPad Prism 9 Statistical Software (GraphPad, San Diego, CA, USA).

## Results

### Running behavior

We monitored the voluntary running behavior of all mice with an automated wheel-attached counter and recorded the turns of the running wheel for each mouse. The animals quickly accepted the running wheel and used it during their wake time at night. We calculated the distance run per day (24 h) and found no difference between the wt animals and the PKP2^+/-^ mice. The average distance run per day during the first 8 weeks of running was 2.56 ± 0.15 km for wildtype littermates (wt) vs. 2.34 ± 0.18 km for PKP2^+/-^ animals (n = 49 and 50, respectively; p = 0.81) and 2.49 ± 0.36 km for wt and 1.86 ± 0.27 for PKP2^+/-^ animals during weeks 9–16 (n = 14 and 16, respectively; p = 0.47).

### Functional and structural development

**Fig 1:**
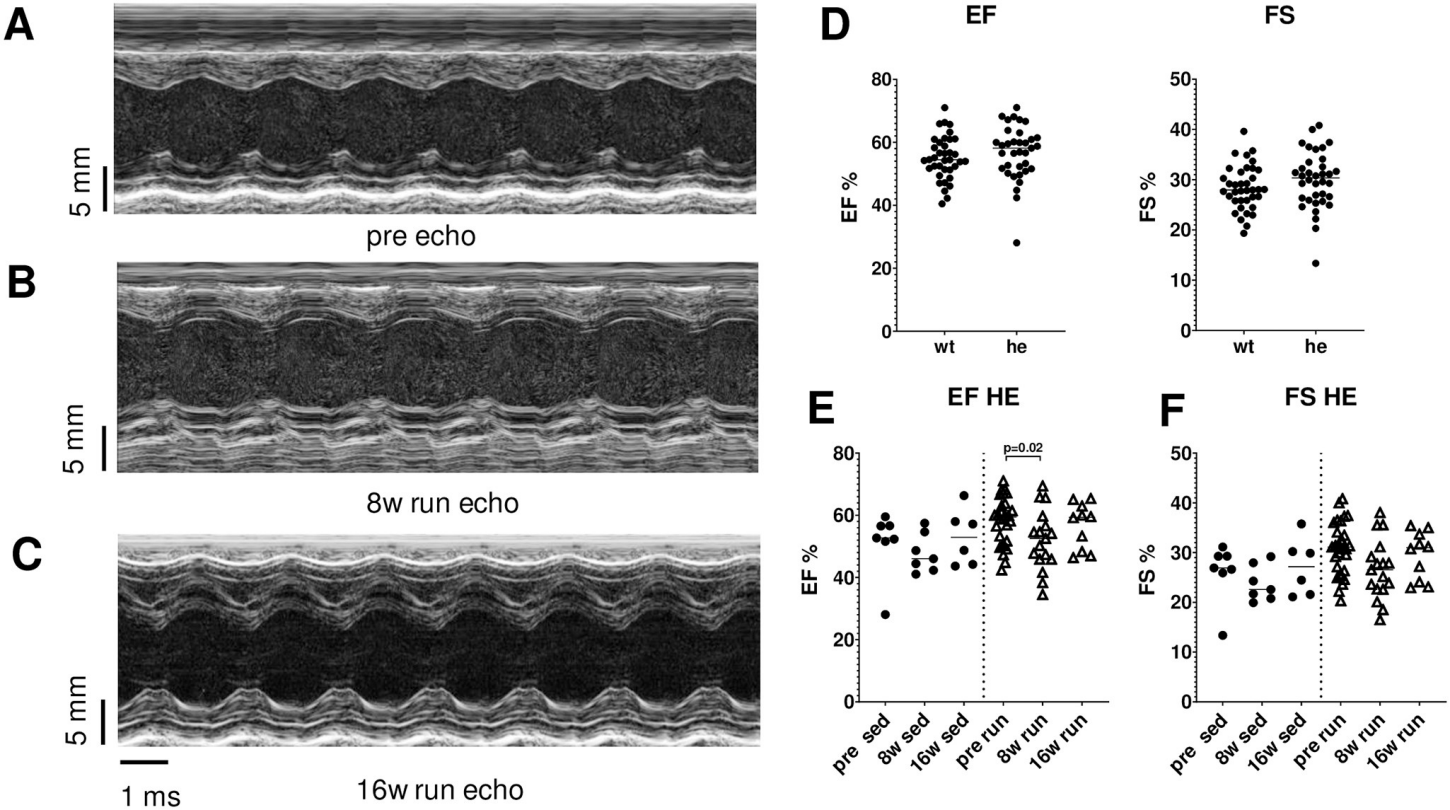
Echocardiographic evaluation of PKP2 mice during 16 weeks of physical activity. Exemplary echocardiographic original traces from mice recorded pre run/sed (**A**), after 8 weeks of running (**B**) and after 16 weeks of running (**C**). There was no difference between the genotypes at baseline in the functional characteristics ejection fraction (EF) and fractional shortening (FS) (**D**). Heterozygous PKP2 mice (he) showed a reduction in EF after 8 weeks of running, which was normalized after 16 weeks (**E**). Heterozygous animals also showed a reduction in FS after 8 weeks of running which normalized again at 16 weeks (**F**).

The echocardiographic study of the mice did not reveal any functional or structural changes due to the heterozygous PKP2 mutation. The values for ejection fraction (EF), fractional shortening (FS), left ventricular inner diameter at diastole (LVID d) and at systole (LVID s) as well as the interventricular septum thickness at diastole (IVS d) and at systole (IVS s) were the same in both genotypes, indicating a very mild form of exercise that did not cause exercise-induced hypertrophy (Table 1 and Fig 1A–1D).

**Table 1:** Echocardiographic analysis.

Parameter (mean ± SEM)	pre sed	8w sed	16w sed	pre run	8w run	16w run
**LVIDs WT**	2.98 ± 0.15	2.91 ± 0.42	3.18 ± 0.09	3.10 ± 0.07	3.15 ± 0,06	3.06 ± 0.12
	n = 11	n = 7	n = 6	n = 25	n = 17	n = 11
**LVIDs HE**	3.40 ± 0.24	3.19 ± 0.11	3.16 ± 0.11	2.94 ± 0.07	3.14 ± 0.13	2.99 ± 0.07
	n = 7	n = 7	n = 6	n = 28	n = 16	n = 10
**LVIDd WT**	4.08 ± 0.11	4.0 ± 0.08	4.18 ± 0.12	4.08 ± 0.06	4.02 ± 0.05	4.04 ± 0.11
	n = 10	n = 7	n = 6	n = 25	n = 17	n = 11
**LVIDd HE**	4.40 ± 0.19	4.09 ± 0.14	4.12 ± 0.11	4.09 ± 0.05	4.06 ± 0.09	4.03 ± 0.09
	n = 7	n = 7	n = 6	n = 28	n = 16	n = 10
**IVSs WT**	1.41 ± 0.09	1.45 ± 0.03	1.43 ± 0.07	1.48 ± 0.04	1.38 ± 0.04	1.41 ± 0.05
	n = 11	n = 7	n = 6	n = 27	n = 17	n = 10
**IVSs HE**	1.37 ± 0.09	1.20 ± 0,05	1.45 ± 0.07	1.43 ± 0.03	1.52 ± 0.04	1.49 ± 0.06
	n = 7	n = 7	n = 6	n = 29	n = 17	n = 9
**IVSd WT**	0.95 ± 0.05	1.03 ± 0.04	1.05 ± 0.04	1.07 ± 0.03	1.07 ± 0.02	1.06 ± 0.04
	n = 10	n = 7	n = 6	n = 27	n = 18	n = 10
**IVSd HE**	0.98 ± 0.08	0.86 ± 0.03	1.04 ± 0.05	1.06 ± 0.03	1.12 ± 0.04	1.09 ± 0.04
	n = 7	n = 7	n = 6	n = 28	n = 18	n = 10

LVIDs = left ventricular inner diameter at systole; LVIDd = left ventricular inner diameter at diastole; IVSs = interventricular septum thickness at systole; IVSd = interventricular septum thickness at diastole.

We compared the data for each genotype individually to calculate whether there was a time-dependent (sedentary, sed) or activity (run)-dependent shift in heart function or structure. We found no time- or running-dependent change in EF and FS in wildtype mice, however, we could detect a change in EF (from 58.1 ± 1.4% to 52 ± 2.4% after 8 weeks, p = 0.02) which returned to normal after 16 weeks of running (56.7 ± 2.3%, p = 0.9 vs. baseline) in PKP2^+/-^ mice as well as in FS (from 30.8 ± 0.9% to 26.6 ± 1.5% after 8 weeks, p = 0.07) also returning to normal after 16 weeks of running (29.5 ± 1.5%, p = 0.9 vs. baseline and p = 0.02 in two-way ANOVA time dependent difference). Such a transient change was not observed in the structural parameters such as LVID d/s and IVS d/s (Table 1). HW/BW was unchanged in both genotypes (6.0 ± 0.18 mg/g in wt, n = 5, vs. 5.9 ± 0.27 mg/ in PKP2^+/-^, n = 6) and was not affected by the physical activity of the animals (wt 8w run: 5.9 ± 0.34 mg/g, n = 8, vs. he 8w run: 5.9 ± 0.35 mg/g, n = 7, and wt 16w run: 5.7 ± 0.38 mg/g, n = 6, vs. he 16w run: 5.3 ± 0.16 mg/g, n = 9).

### Electrophysiology *in vivo*

The electrophysiological study in mice showed inducibility of arrhythmias in 18% of wildtype mice at baseline (sed, n = 11), which was only slightly increased after 8 (n = 7) and 16 weeks (n = 4) of running (to 29% and 25%, respectively (p = 0.605 sed vs. 8 weeks and p = 0.770 sed vs. 16 weeks). An acute additional ISO challenge caused a slight increase of inducibility in sedentary mice (27% inducibility), which was further increased after 8 and 16 weeks of running to 57 and 50%, respectively (p = 0.205 sed vs. 8 weeks and p = 0.409 sed vs. 16 weeks).

**Fig 2:**
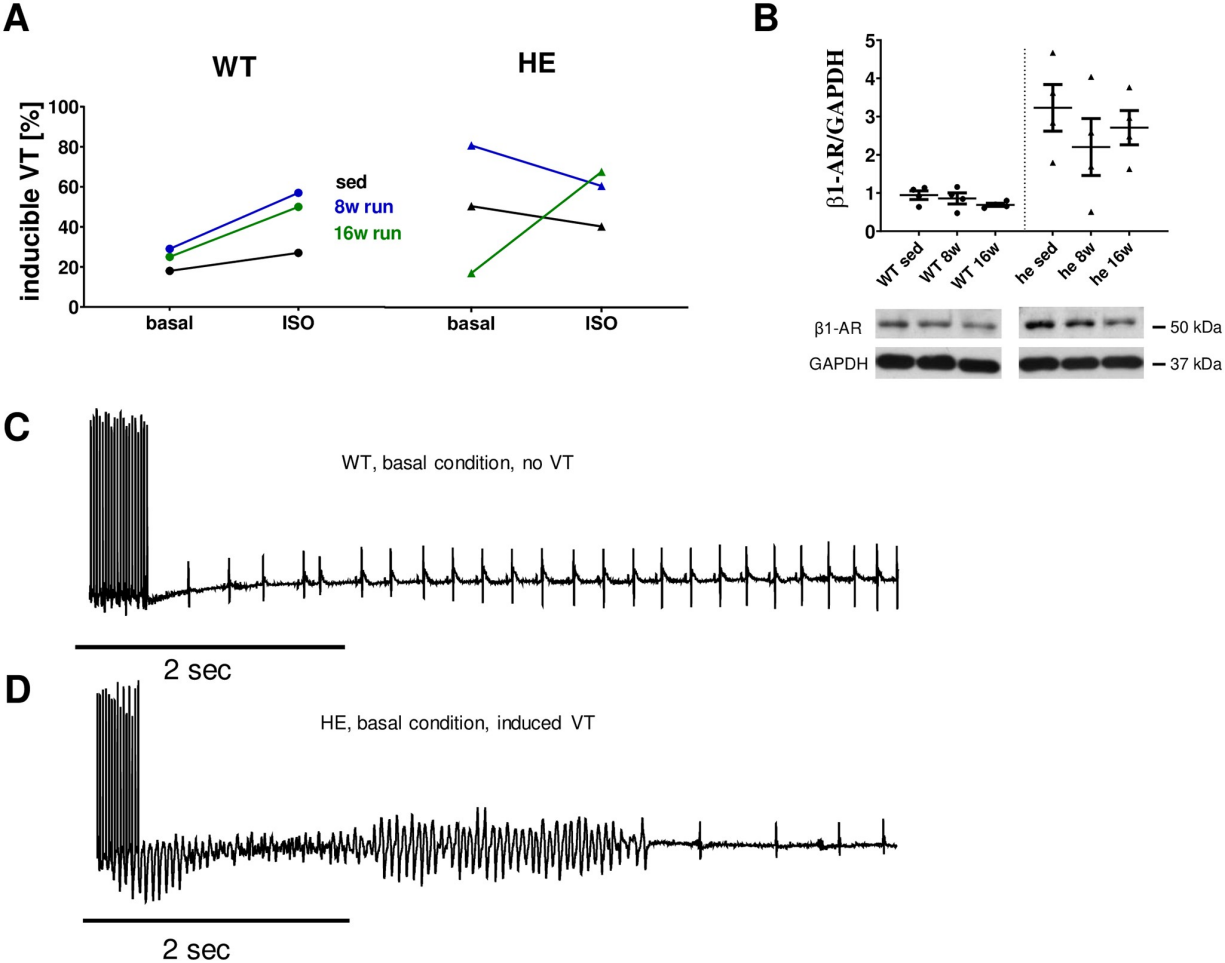
Electrophysiological in vivo study of mice after long-term training. (**A**) The electrophysiological study revealed that long-term training in mice did not cause an increased vulnerability in wt mice under basal conditions (18% in sedentary animals, n = 11 vs. 29% after 8 weeks, n = 7 and 25% after 16 weeks of running, n = 4). An additional ISO challenge did cause a slightly increased occurrence of arrhythmia in sedentary mice from 18% at basal conditions to 27% under ISO, which was increased to 57% under ISO after 8 weeks of training. This vulnerability was reduced after 16 weeks of training to 50%. The heterozygous animals showed increased occurrence of arrhythmia under basal conditions in sedentary mice of 50% (n = 10), which even increased after 8 weeks of training (80%; n = 5), but was reduced to only 17% after 16 weeks of training (n = 6). Interestingly the additional ISO challenge did not cause an increase of arrhythmia in heterozygous animals at rest or after 16 weeks of training. After 8 weeks of training the rate of arrhythmia observed dropped slightly. B Protein expression levels of β1-adrenergic receptor in cardiac tissue of wt mice was unaffected by voluntary running but was significantly lower compared to PKP2^+/-^ mice (0.942 ± 0.113 vs. 3.229 ± 0.609, respectively; p = 0.0181). C representative traces from wt sedentary mice under basal conditions without arrhythmia and D from PKP2^+/-^ sedentary mice under basal conditions displaying arrhythmia after burst stimulation.

In contrast to these findings, inducibility in sedentary PKP2^+/-^ mice was increased at baseline (50% inducibility, n = 10, p = 0.122 vs. wt sed). The number of sedentary PKP2^+/-^ mice showing induced arrhythmias upon ISO was unaltered compared to WT (40% inducibility, p = 0.653). After 8 weeks of running, the inducibility was increased to 80% (n = 5, p = 0.264 vs sed), which was numerically reduced to 60% after ISO challenge (p = 0.490 vs basal). Again, this drop might in part be due to the lower number of animals studied after 8 weeks of running compared to the sedentary animals. Surprisingly, after 16 weeks of running, only 17% of PKP2^+/-^ mice (n = 6) had inducible arrhythmias, which was increased to 67% by ISO (p = 0.079 vs. basal; Fig 2**A**).

To evaluate potential mechanisms leading to a higher propensity for arrhythmias in PKP2^+/-^ mice at rest, we measured the protein levels of β1-adrenergic receptor (β1-AR). β1-ARs regulate contractility and PKA-mediated effects on various signaling proteins in cardiac myocytes but can cause detrimental effects when stimulated chronically [25]. The relative protein level of the β1- AR was markedly higher in PKP2^+/-^ mice compared to their wt littermates (3.23 ± 0.61 vs. 0.94 ± 0.11, p = 0.018; Fig 2**B**), however, there was a greater scatter within the tested PKP2^+/-^ mice. The protein level was unaffected by voluntary running in both genotypes with a slight decrease in the PKP2^+/-^ mice.

### Cellular Ca-handling and contractility

To test whether abnormal cellular Ca cycling could explain some of the changes observed in functional alterations, we analyzed cellular Ca handling in isolated cardiac myocytes and found slight time-dependent alterations after long-term activity in PKP2^+/-^ mice.

**Fig 3:**
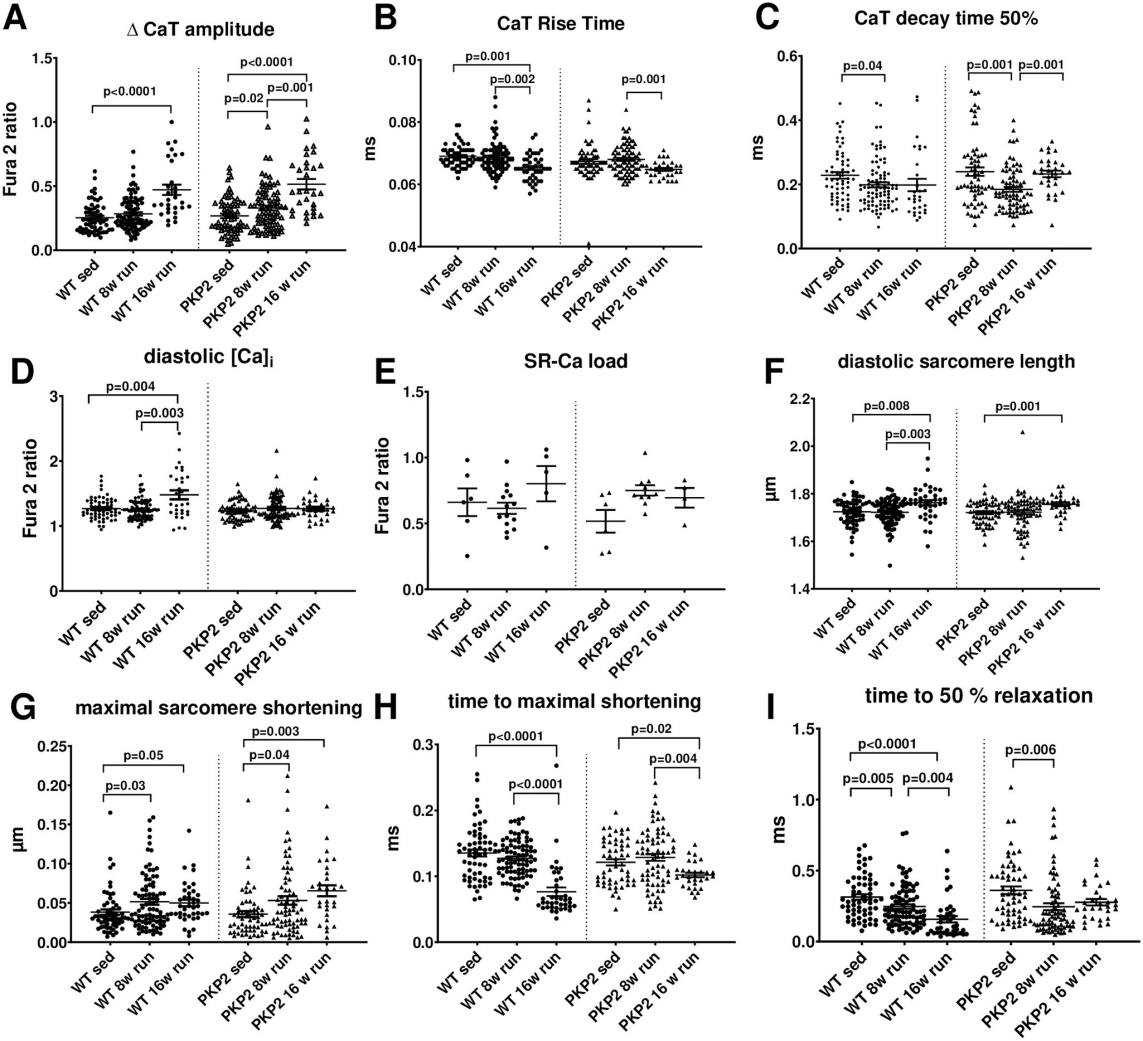
Cellular Ca cycling and contractions after long-term training. Fura-2 loaded cardiac myocytes were electrically stimulated and CaT measured. The CaT amplitude significantly increased after 16 weeks of training in both genotypes with p<0.0001 (**A**). While the CaT rise time was significantly faster in cells from wt mice after 16 weeks training, this trend was already significant in cells from the PKP2^+/-^ animals after 8 weeks (**B**). However, the decay time to reach 50% of the maximal amplitude did change significantly in wt mice during 8 weeks of training (p = 0.04), while the PKP2^+/-^ mice showed a significant (p = 0.001) acceleration after 8 weeks of running that was abolished again after 16 weeks (**C**). Diastolic Ca values did not change in PKP2^+/-^ mice during the course of training after 8 and 16 weeks, but the wt had a distinct increase in diastolic Ca values after 16 weeks of training (p = 0.004) (**D**). Conversely, the SR Ca load measured by caffeine application, was unchanged in PKP2^+/-^ mice and in wt animals (**E**). In parallel to the fluorescent measurement of cytosolic Ca, we recorded the contractility by measuring the sarcomere length with a bright field video camera system. The diastolic sarcomere length was longer after 16 weeks of training in wt animals (p = 0.008), albeit the diastolic Ca levels were increased (**F**). In accordance to the CaT amplitudes, the maximal shortening of the cells is significantly increased in PKP2^+/-^ mice after 16 weeks of training (p = 0.0023), however this translation was not observed in wt animals were only a slight but non-significant increase was seen (**G**). Time to maximal shortening was significantly faster in wt mice after 8 (p = 0.03) and 16 weeks of training (p = 0.05) and in PKP2^+/-^ animals, as seen in Ca kinetics (**H**). The time to 50% relaxation, was significantly faster after 8 weeks of training in PKP2^+/-^ mice (p = 0.006) but not after 16 weeks (**I**). All data are shown as single cell data points from different animals: wt sed: 4 animals, wt 8 weeks: 6 animals, wt 16 weeks: 3 animals, hw sed: 4 animals, PKP2^+/-^ 8 weeks: 5 animals and PKP2^+/-^ 16 weeks: 2 animals).

We analyzed CaT in cells loaded with Fura2 and found that the cells from wildtype animals developed an increased diastolic Ca concentration after 16 weeks of physical activity, which also translated into a significantly enlarged peak Ca release from the SR during each cardiac cycle (Fig 3**A** and 3**D**). While this enlarged CaT amplitude was also observed in cells from PKP2^+/-^ mice after 16 weeks of training, an increased diastolic cytosolic Ca concentration could not be observed. It has to be noted that baseline values for diastolic and systolic Ca concentration as well as the time course before training were equal between the different genotypes. Interestingly, the CaT duration (CaTD), a well-established factor influencing the propensity for arrhythmias [23, 26] showed small but distinct differences between the genotypes. CaT rise time was markedly faster after 16 weeks of training, indicating a potential adaptation to the physical activity and related higher demand. A similar shift could not be detected in the cells from the PKP2^+/-^ mice (Fig 3**B**). In contrast, the decay times were not affected in wildtype animals, where the times remained constant even after 16 weeks of physical activity. The PKP2^+/-^ mice instead showed a markedly fastened decay rate after 8 weeks of training (contrary to the rise time) which returned to initial values after 16 weeks (Fig 3**C**). Taken together, wt animals showed shortened CaTD after 16 weeks of voluntary training (p = 0.018 vs. baseline) which could not be observed in PKP2^+/-^ mice which had a transient CaTD shortening after 8 weeks of training (p = 0.022 vs. baseline). SR Ca content showed a tendency towards higher content after 16 weeks of training in wildtype animals, albeit not statistically significant. This change was not observed in PKP2^+/-^ mice where SR Ca content remained constant (Fig 3**E**). The calculated fractional release (FR) from these mean data indicates a higher FR after 16 weeks of training in both wt and PKP2^+/-^ animals.

The cellular contractility is impaired in PKP2^+/-^ mice after long term activity with a time dependent development. The contractility of cardiac myocytes is directly affected by the Ca cycling, as Ca acts as the main second messenger between cardiac excitation and the actual contraction [27]. We recorded the sarcomere shortening after electrical stimulation to have a measure of the contractility of the single cells. Despite the increased diastolic Ca concentration in wildtype cells after 16 weeks of training, we found a longer sarcomere length compared to the initial values, indicating a stronger relaxation during diastole (Fig 3**F**). This was also seen as a trend in PKP2^+/-^ mice (p = 0.075). Interestingly, the maximal shortening in wildtype cells was not increased in wildtype cells even though the systolic Ca was higher after 16 weeks of training (albeit a trend towards higher values was detected). In contrast, the PKP2^+/-^ cells showed a larger contraction after 16 weeks of training, in accordance with the systolic Ca (Fig 3**G**). The time until the maximal contraction was reached was significantly faster after 16 weeks of training in wildtype but not PKP2^+/-^ cells (Fig 3**H**). We were however surprised to find a distinct fastening of the relaxation time after 16 weeks of training in wildtype mice, which was not visible in the CaTs. The PKP2^+/-^ cells had a quicker relaxation after 8 weeks, which was not maintained after 16 weeks of training (Fig 3**I**).

#### Histology

**Fig 4:**
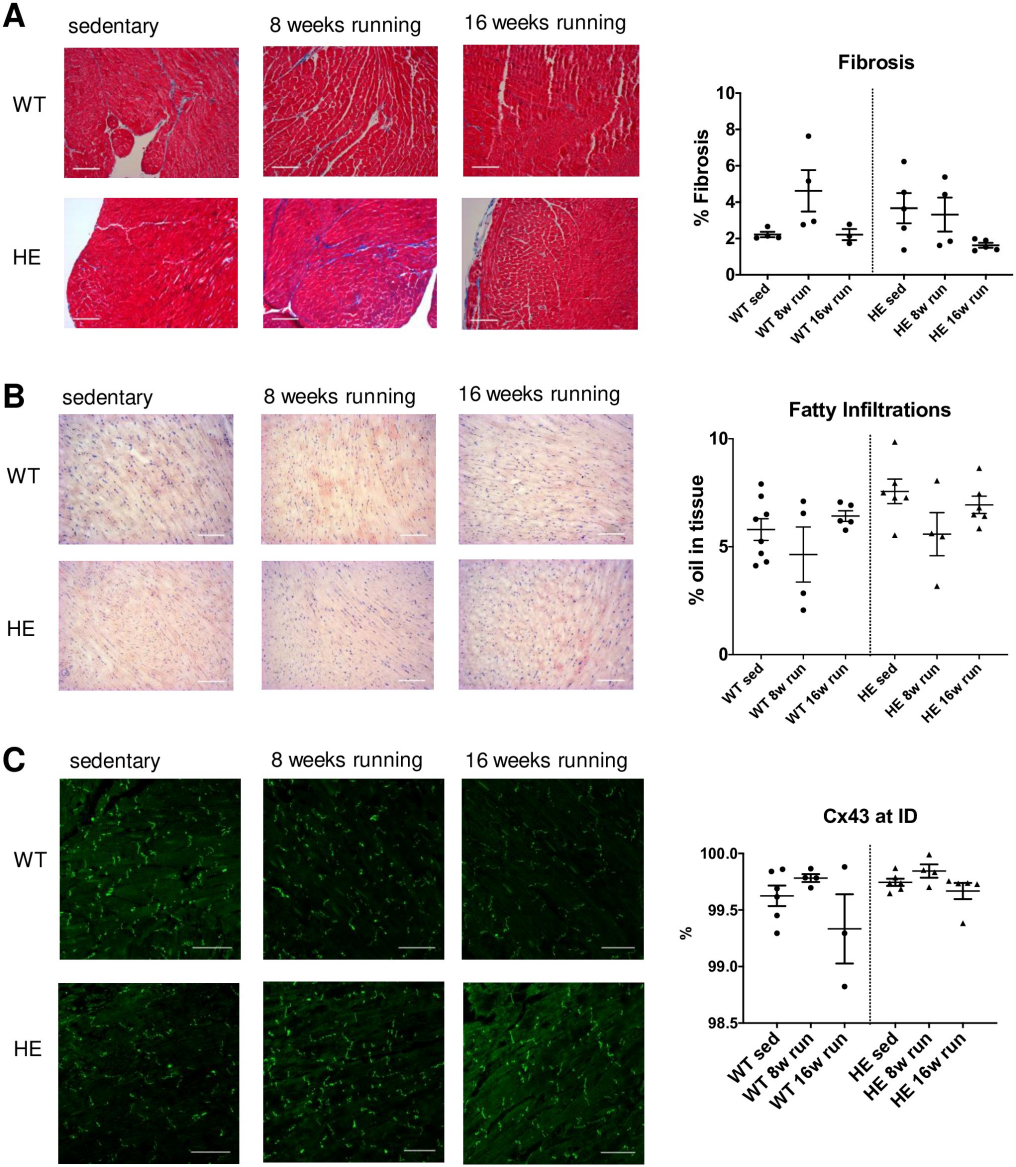
Histological analysis of cardiac tissue after long-term training. (**A**) Representative images for sedentary, 8 weeks of running and 16 weeks of running tissue from wt (upper row) and PKP2^+/-^ mice (lower row) are shown for Trichrome-stained ventricular slices. No change of fibrous tissue could be detected in either genotype or in response to long-term training. (**B**) The oil stain in frozen sections from cardiac tissue with oil red did not reveal an increase in fatty replacements. (**C**) We labeled the gap junctions with a Cx43 specific antibody and analyzed the images by confocal microscopy. The connexins were predominantly located at the intercalated discs as seen in the representative images shown. There was no shift in subcellular distribution due to genotype or long-term training observed. The single data points indicate the number of animals analyzed.

Fibro-fatty replacements are often found in ARVC patients during disease progression [28, 29], we thus studied the composition of cardiac tissue slices from sedentary and running mice. The histological analysis of cardiac tissue sections taken from the transverse plane did not show an increased amount of fibrosis after long-term training in PKP2^+/-^ mice. Ventricular slices were stained with Tri-Chrome-Masson to analyze the relative amount of fibrosis in each slice. We could not observe a discernable difference between the genotypes and the long-term physical activity did not cause an in- or decrease in fibrosis in neither the wildtype nor the PKP2^+/-^ mice. Furthermore, we could not detect any differences between the left and right ventricular areas (Fig 4**A**).

Moreover, the oil red staining did not reveal a significant increase in fatty replacement in the cardiac tissue due to the physical activity. However, we found a higher degree of fatty infiltration in the PKP2^+/-^ mice at baseline condition before running in comparison to the wildtype animals (7.6 ± 0.57% vs. 5.8 ± 0.50%; p = 0.039, unpaired t-test; Fig 4**B**).

Since gap junctions are linked to the desmosomes through PKP2 [30], we analyzed the localization of the building blocks of gap junctions, Cx43, in the cells to understand whether a mislocalization could cause a potential loss of functional channels at the intercalated disc (ID). When calculating the amount of Cx43 labeling at the ID relative to the cytosolic localization, we found no shift of Cx43 away from the intercalated discs (ID) after long-term training or due to the genotype (Fig 4**C**). To verify that the Cx43 was indeed localized at the intercalated disc, we performed double labeling of the slices with N-cadherin, which is a specific marker for the IDs and found a high overlap of both labels (S3 Fig in S1 File).

### Protein expression

**Fig 5:**
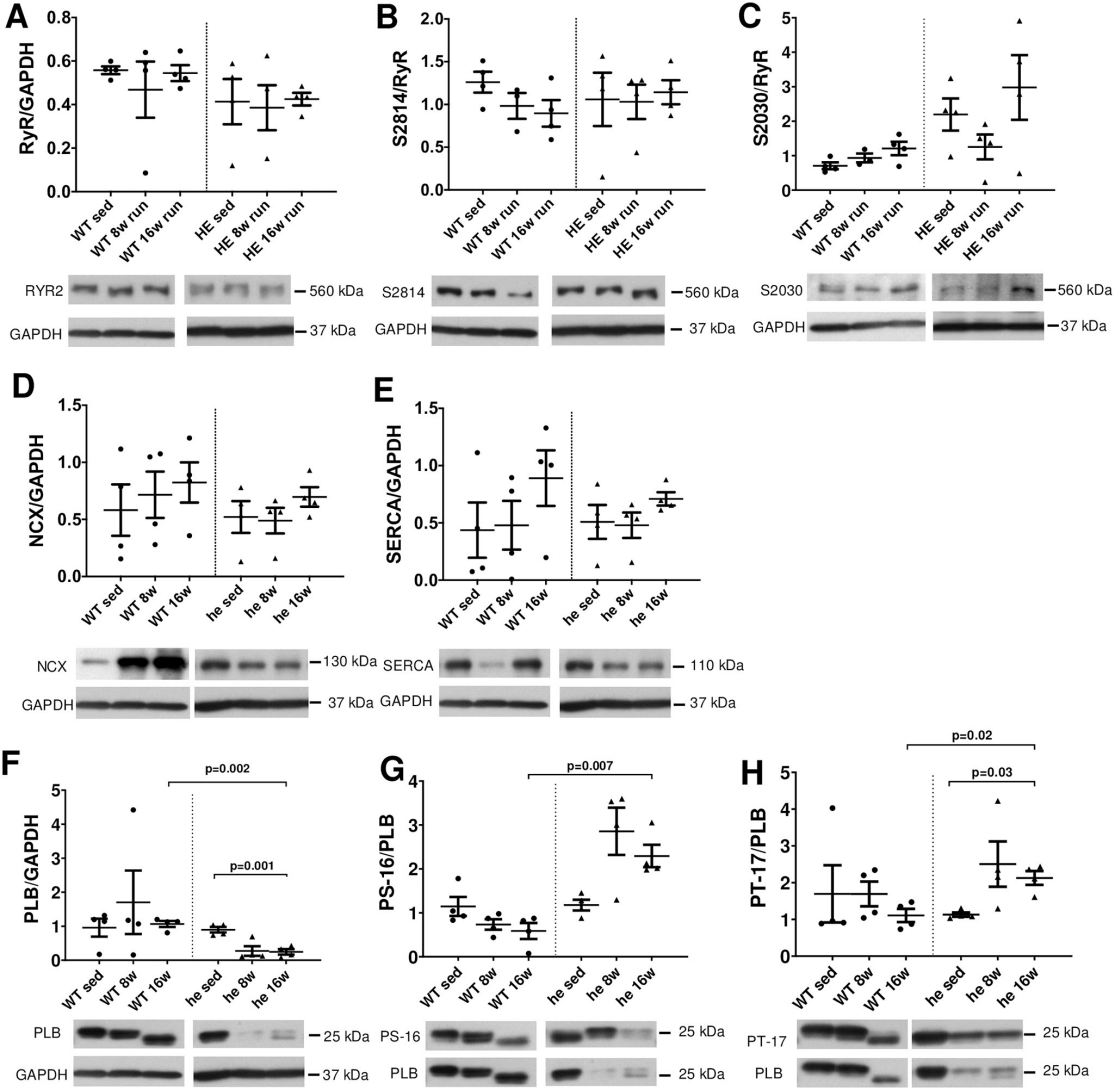
Protein expression levels of ECC key players. We analyzed the protein expression levels by Western Blot to reveal the potential changes underlying the functional alterations we have found in cells from wt and PKP2^+/-^ mice with and without training. First, we analyzed the RYR2 protein levels and found no changes neither between the genotypes nor due to training (**A**). In addition, the phosphorylation of the RyR by CaMKII at S2814 is not affected (**B**) but the PKA mediated phosphorylation at S2030 is higher in PKP2^+/-^ animals but unaffected by training (**C**), indicating that the Ca release from the store is unaffected by physical activity. The protein level of the Na-Ca-exchanger NCX is also unchanged and thus most likely does not play a significant role in shifted Ca removal processes (**D**). In accordance with the SR-Ca load data, we found a slight shift towards higher expression of SERCA protein in wt animals after 16 weeks of training, albeit not significant (**E**). The overall PLB protein level as well as the PLB-phosphorylation was unchanged in wt animals (**F-H**), whereas the PLB protein level was significantly reduced in PKP2^+/-^ animals after 16 weeks (p = 0.001) of training. In addition to the protein levels of PLB, the phosphorylation by PKA at Ser16 was significantly increased after 16 weeks of training (p = 0.007) in PKP2^+/-^ mice compared to wt mice after 16 weeks (**G**). A similar pattern was found at the phosphorylation site for CaMKII, Thr17, which was significantly increased as well (p = 0.03) after 16 weeks, as well as in comparison to wt animals after 16 weeks (p = 0.03) (**H**).

To test whether changes on the protein level might be the underlying cause for the functional changes we observed on the cellular level (or lack of thereof in HE mice such as CaTD shortening, diastolic Ca levels) we performed extensive Western Blot analyses. First, we compared the protein expression levels of ECC key players at baseline without physical activity in the two different genotypes. We found that the analyzed proteins were not changed due to genotype (RyR, SERCA, PLB, NCX as well as the phosphorylation of RyR at S2814 and PLB at pS-16 and pT-17; S2 Fig in S1 File), except the PKA-mediated phosphorylation of the RyR at S2030, which was significantly increased in he mice (p = 0.008; Fig 5**C**). To get a better understanding of molecular changes that might occur during long-term training, we compared the protein levels of ECC key players after 8 and 16 weeks of running. We found that RyR protein level in wt mice or in PKP2^+/-^ mice after 8 and 16 weeks (Fig 5**A**) and the phosphorylation of the RyR by CaMKII at Ser2814, as often found during hypertrophic signaling, was not affected by training (Fig 5**B**). This indicates that the altered diastolic Ca level in wt mice after training could not be accounted to the protein level or CaMKII-dependent regulation of the Ca release channel. However, we found higher PKA-mediated phosphorylation of the RyR at S2030 in PKP2^+/-^ mice, which remained unaltered after 8 or 16 weeks of running (Fig 5**C**). We then measured the protein level of the Na-Ca-exchanger NCX and again found no shift due to the training in both genotypes, indicating that the NCX protein level does not play a role in the observed changes in Ca cycling (esp. Ca removal; Fig 5**D**). However, the SERCA protein level showed a trend towards higher protein levels in wt mice after 16 weeks of training, which might explain the slightly increased SR Ca content seen in wt mice (Figs 3**E** and 5**E**). Interestingly, total PLB protein level was unchanged in wt mice during training, but was significantly reduced in PKP2^+/-^ mice after 16 weeks of training, pointing towards a shift in SERCA regulation (Fig 5**F**). Similarly, the phosphorylation of PLB by PKA at Ser16 and CaMKII at Thr17 was significantly increased after 8 weeks of training, however, in both cases the phosphorylation level was partially reduced after 16 weeks of training (Fig 5**G** and 5**H**).

## Discussion

ARVC is a hereditary disease with its disease-causing mutations mostly located in desmosomal proteins of which PKP2 has the highest incidence [2] among all identified ARVC cases. However, risk stratification remains difficult, and often includes previous cardiac events such as recent syncope or non-sustained VT [6] and thus is not useful for family members that are asymptomatic at the time of diagnosis. In fact, 88% of diagnosed ARVC patients have multiple mutations [1] and exercise is considered a major environmental factor influencing the disease progression of ARVC. To date there is no consensus on permissible exercise for ARVC patients and their family members and results are contradictory [31] where some groups found that endurance exercises increase the disease severity [13] and others report that only sports with a highly dynamic component, increasing oxygen uptake by more than 70% is detrimental [14, 32]. Ruwald et al. [15] on the contrary found that recreational sports, even highly dynamic, do not increase the arrhythmogenic risk while Mazzanti et al. [33] report an increased risk already at fairly modest levels of exercise. These contradictory findings and the importance of treatment guidelines for patients and asymptomatic family members alike call for controlled studies on the subject.

There are various animal models available to mimic ARVC with and without physical exercise [34]. Specific mouse models with a PKP2 mutation have shown that a homozygous knockout of PKP2 is lethal at an embryonic stage [19] and the heterozygous mutation does not develop overt structural alterations [21]. In fact, we could confirm a lack of overt structural changes in the mouse model at rest and did not find an increased arrhythmogenic potential *in vivo* as seen in our electrophysiological study. In addition, structural and functional *in vivo* data did not show any signs of deterioration in sedentary mice and also *in vitro* we could not find any alterations in function as measured by Ca handling and contractility as well as protein expression or structure of the cardiac tissue. This is in line with the notion that not all patients carrying a PKP2 mutation develop symptoms and, as elaborated above, additional triggers may be needed to induce arrhythmogenic disease progression.

Although inducible homozygous mutations of PKP2 do induce pathogenic alterations in a mouse model [20, 35] they might not reflect the situation in patients properly. Exercise models are needed to understand the influence of exercise on disease progression and several groups have tested different models in the past carrying plakoglobin or desmoplakin mutations [34] but also an AAV-induced PKP2 knockout model that has been exercised has been described [36]. Recently, a heterozygous PKP2 knockout mouse was exercised on a treadmill and the effects on *in vivo* and *in vitro* structure and function have been described [37]. They could not find an exaggerated phenotype after physical exercise as measured by protein expression levels of key players such as Ca_V_1.2 or AnkB or fibro fatty deposits in the heart tissue. They did instead detect a slowed conduction velocity in the right ventricle and an increased susceptibility for VT *ex vivo*.

However, all these exercise models use different approaches and often include additional stressors such as electric shocks or forced swimming, of which both may cause additional stress that might exacerbate the phenotype described. Also, the duration of endurance training might play a critical role, we thus decided to expose our animals to 8 and 16 weeks of voluntary exercise. Even after 16 weeks of training, we could not observe any structural changes by echocardiography nor by histological techniques. We speculate that this form of exercise might mimic light leisure exercise.

The lack of fibro fatty replacements or relocation of Cx43 even after 16 weeks of training is in line with the finding that PKP2-induced electropathy can occur independent of structural perturbations but might still be causative for SCD in patients [38]. Although fibrofatty replacements are often found in ARVC patients this is not an exclusive trait to the disease. It has to be noted that we monitored left ventricular structure and function and might thus have missed early changes of the right ventricle.

However, we did see a mildly reduced ejection fraction and fractional shortening in PKP2 mice after 8 weeks of training. Interestingly, this reduction was abolished after 16 weeks of training. We speculate that the animals were able to adapt to the light exercise regimen after an initial period of maladaptation. This would point towards a beneficial effect of light voluntary training on cardiac function in predisposed animals in the long term. The lack of structural alterations in conjunction with functional changes can also be seen in ARVC patients with a PKP2 mutation who do not necessarily show structural alterations when electropathy develops [38]. Furthermore some reports indicate a risk reduction due to regular physical activity [39]. On a cellular level we observed complex changes in Ca handling that are not easy to link to pro-arrhythmic activity. Despite unchanged SR load, we observed a significant increase in Ca transient amplitude for both trained WT and trained mice with heterozygous PKP2 loss-of-function. Interestingly, the Ca rise time was also significantly faster for both trained genotypes indicating that training stimulated coupled-gating from LTCC to RyR2 by either increased LTCC or enhanced fractional RyR2 release. We have evidence for the latter, since calculated fractional release was increased after 16 weeks of training in both wt and PKP2+/- animals. The effects of training on diastolic function are even more complex but may include an enhancement of SERCA function and increased diastolic SR Ca leak. We did not measure the latter parameter directly, but it can be inferred from the Ca transient kinetics and SR Ca load data. Interestingly, this training effect on diastolic Ca handling was substantially different between WT and PKP2+/- animals: While enhanced SR Ca leak in WT with moderate stimulation of SERCA may have resulted in increased diastolic Ca concentration at least after 16 weeks of running, a more pronounced stimulation of SERCA in PKP2+/- animals may have prevented the increase in diastolic Ca.

Our data stand in contrast to recent findings by van Opbergen et al. [37], who could detect changes due to voluntary exercise as soon as 4 weeks after the onset of training. However it has to be pointed out, that their animal model already had distinct changes at rest, which we could not detect. Also, it is unclear whether the functional changes presented after training in the aforementioned study can be solely attributed to the exercise regimen or might in fact be caused by the genotype itself. The alterations we found on the protein level cannot fully explain the changes in Ca handling. Interestingly, we found a significantly increased PKA-mediated RyR2 phosphorylation at S2030, a phosphorylation site that is crucial for a complete and adequate β-adrenergic response in cardiac myocytes [40]. A recent report by Wang et al. [41] found that this site is hyperphosphorylated in left ventricular tissue of ARVC patients and they went on to show that a S2030 hyperphosphorylation causes DADs and spontaneous Ca release in a mouse model. Follow up studies will be necessary to understand the role of the L-type Ca channels in our model as altered Ca currents might give an explanation for the changes we see in CaT amplitude. Localization and number of RyRs within a couplon might also influence the amount of Ca release from the SR during a twitch.

In order to understand the actual effect of physical exercise on arrhythmogenity, we performed electrophysiological studies *in vivo*. These studies led to two interesting findings: first in wt animals the arrythmogenic potential under baseline conditions is unaffected by long-term training. However, an additional ISO challenge increased the risk in trained animals, which becomes detectable already after 8 weeks and is partially reduced after another 8 weeks of training. A study on rats could show that long term training could induce arrhythmogenic events, even though their training model potentially added an additional stressor as the treadmill running was forced [17]. In contrast, we found an increased propensity towards arrhythmia in PKP2^+/-^ mice already at baseline, which is in line with our finding of increased β-AR protein expression in PKP2^+/-^. Here, the long-term running of 16 weeks seemed to be even beneficial as the rate of adverse events dropped.

There was a highly interesting discovery when we tested for arrhythmia under β-adrenergic stimulation. In contrast to the control animals, the PKP2^+/-^ mice did not react at all to the additional stressor, even though the β-AR protein levels remained high in our animals. In contrast to these findings are data from a subset of ARVC patients who have significantly downregulated myocardial β-adrenergic receptors [42, 43]. This discrepancy might be explained by an uncoupling from G_S_ due to the physical exercise, similar to findings in heart failure [44–46]. In addition, an increase in G_α_ might be antagonizing the β-adrenergic signaling in these mice [47–49]. However, the ability to respond to stress by positive inotropy is crucial for a proper stress response and a lack thereof can cause a severely aberrant cardiac phenotype as seen in chronic heart failure [25, 27]. We speculate that the altered response to β-adrenergic signaling might not be caused by the signal itself but more likely by changes in receptor expression and the responsiveness of the RyRs. As discussed above, the hyperphosphrylation at S2030 might be one if not the reason for a reduced responsiveness.

The shift in protein expression that is overt in PLB expression and the highly increased phosphorylation of the almost diminished PLB protein that is left after 8 weeks of training might be indicative of an effort to counterbalance the reduced ability to properly respond to beta adrenergic stimulation. However, further studies will have to be performed to prove this theory, especially since these changes are not clearly reflected in the functional data.

Taken together, our data indicate that low intensity workout does not cause an increase of sudden cardiac death and overt arrhythmia in a mouse model and seems to be even beneficial during an early, transient phase. The inability to respond to β-adrenergic stimuli is indicative for an early phenotype during ARVC disease progression. This model might be useful to shed light on the benefits of light, recreational training for ARVC patients and their family members with no or light symptoms. We conclude that recreational exercise in a mouse model might even be protective and thus a closer look at exercise behavior in asymptomatic or lightly symptomatic mutation carriers might be useful to improve the risk assessment and training guidelines for ARVC patients and their family members. Future studies will have to focus on the effect of additional stressors like acute psychological stress, inflammatory processes or additional mutations.

## Supporting information

S1 File (DOCX)

S1 Raw images (PDF)
