# Supplementary material for: Beneficial effect of voluntary physical exercise in Plakophilin2 transgenic mice
Source: PLoS One. 2021 Jun 4;16(6):e0252649. doi: 10.1371/journal.pone.0252649 (PMC8177441; doi:10.1371/journal.pone.0252649)
Supplement: S1 File — (DOCX) [file pone.0252649.s001.docx]

**S1 File**

Beneficial effect of voluntary physical exercise in Plakophilin2 transgenic mice

Karin P. Hammer, Julian Mustroph, Teresa Stauber, Walter Birchmeier, Stefan Wagner, Lars S. Maier

1. **Western Blot data**

All samples from a genotype (wt or he) were blotted on the same gel to ease the analysis of the activity effect on the protein expression levels. To facilitate the comparison between genotypes, we run reference samples on both gels. All data were normalized to these samples and statistical evaluation was performed on normalized data. The gels were cut after protein separation and incubated with the respective antibodies according to the expected protein size and subsequently analyzed. The original gels are shown below (Suppl Figs 1&2).

The intensities of the specific bands were normalized by their respective housekeeping band (GAPDH) and subsequently normalized to the reference samples indicated to compare samples across gels.

1. **Connexin localization**

We analyzed the relative localization of Cx43 within the cells in tissue slices from the mouse hearts. To assure that the detected signal was indeed localized at the intercalated disc, we performed a double labeling of Cx43 with N-cadherin, which is exclusively localized at the intercalated disc. We then visualized the two proteins by color-coding the co-localized pixels in each image as white color. The images show a high overlap between the two proteins and confirm the predominant localization of Cx43 at the intercalated disc (Suppl. Fig. 3).


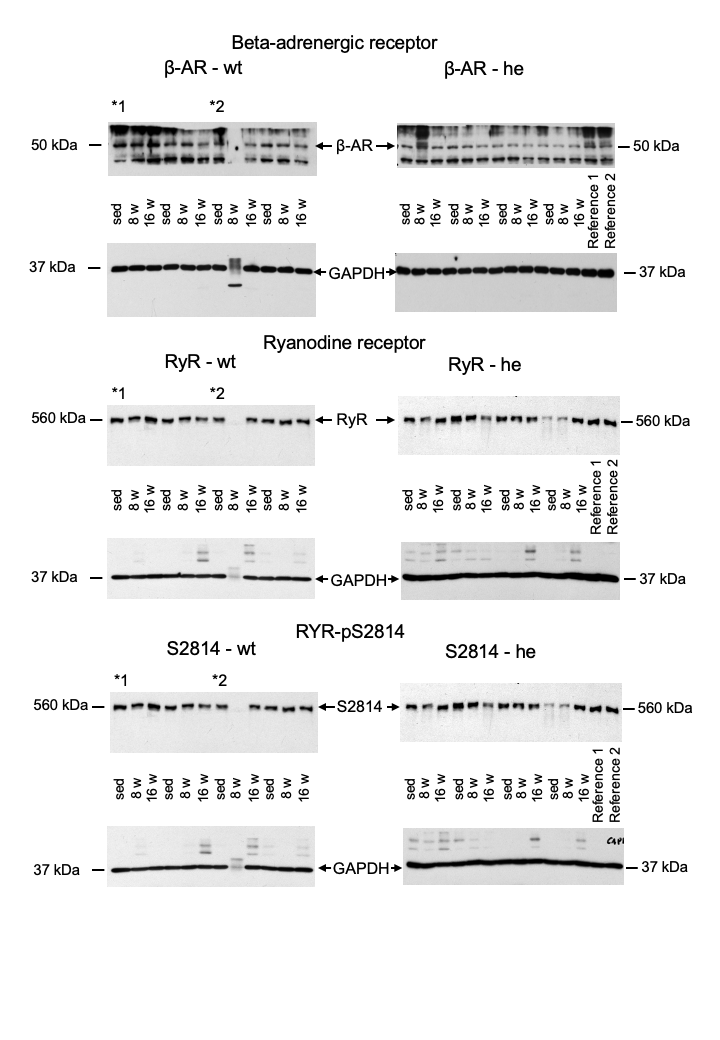


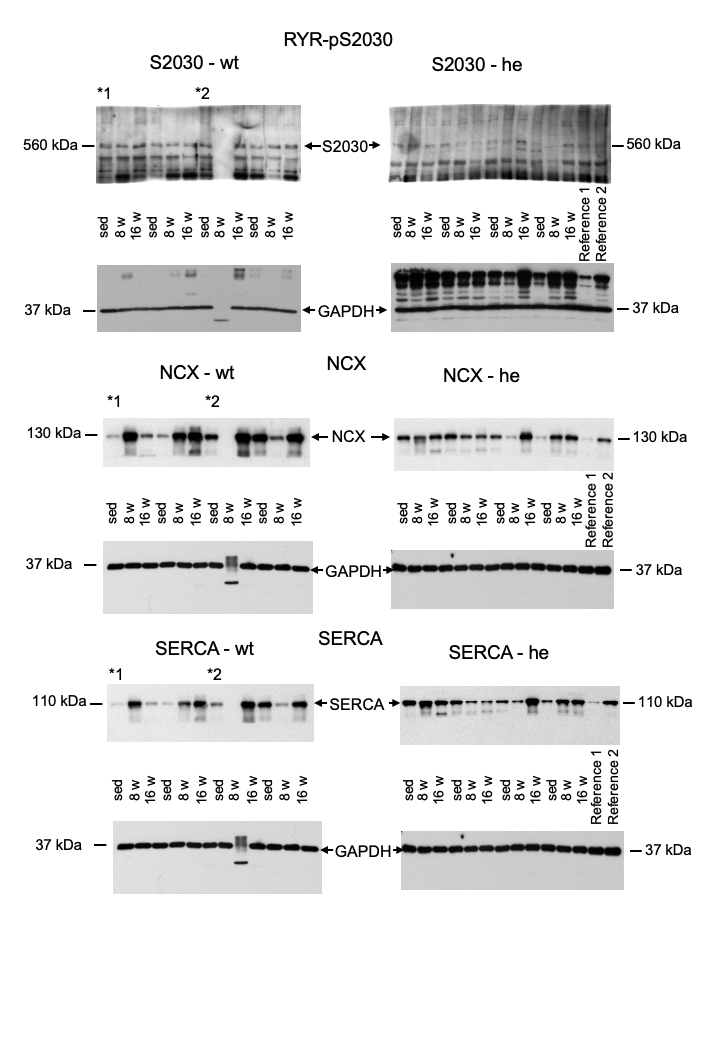


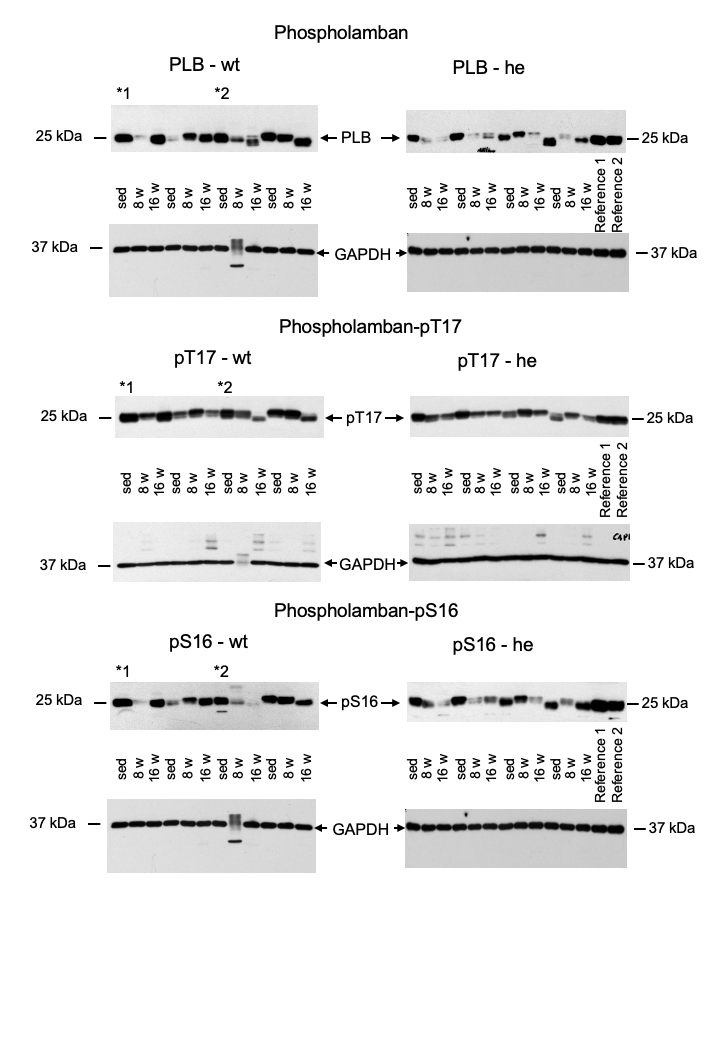


**S1 Fig: Original Western Blot gels.** The original gels were used to calculate the relative protein expression levels. For all proteins data from wt animals and PKP2^+/-^ animals were blotted on separate gels. To compare the genotypes, two reference samples were run on both gels for each protein. The samples chosen were taken from sedentary wt animals, indicated as *1 and *2 for reference 1 and reference 2, respectively. The analyzed lane and protein size is indicated for each gel.


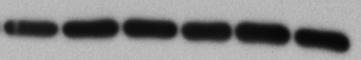

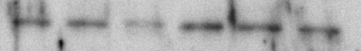


β-AR

GAPDH

37 kDa

50 kDa

wt sed

wt 8 w

wt 16 w

he sed

he 8 w

he 16 w


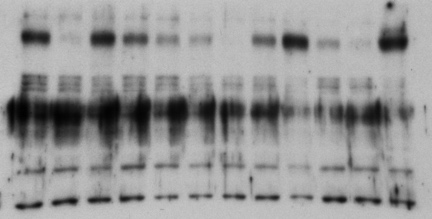

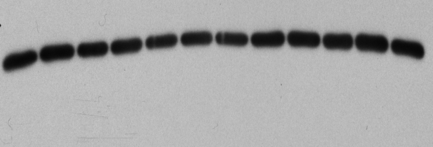


37 kDa

GAPDH

β-AR

50 kDa

wt sed

wt 8 w

wt 16 w

he sed

he 8 w

he 16 w

wt sed

wt 8 w

wt 16 w

he sed

he 8 w

he 16 w

**S2 Fig: Genotype comparison of β-AR.** The original gels were used to calculate the relative protein expression levels. To directly compare the genotypes, a subset of samples from both genotypes were blotted on the same gel. This direct comparison confirms the method described above.

8w

16w

sedentary


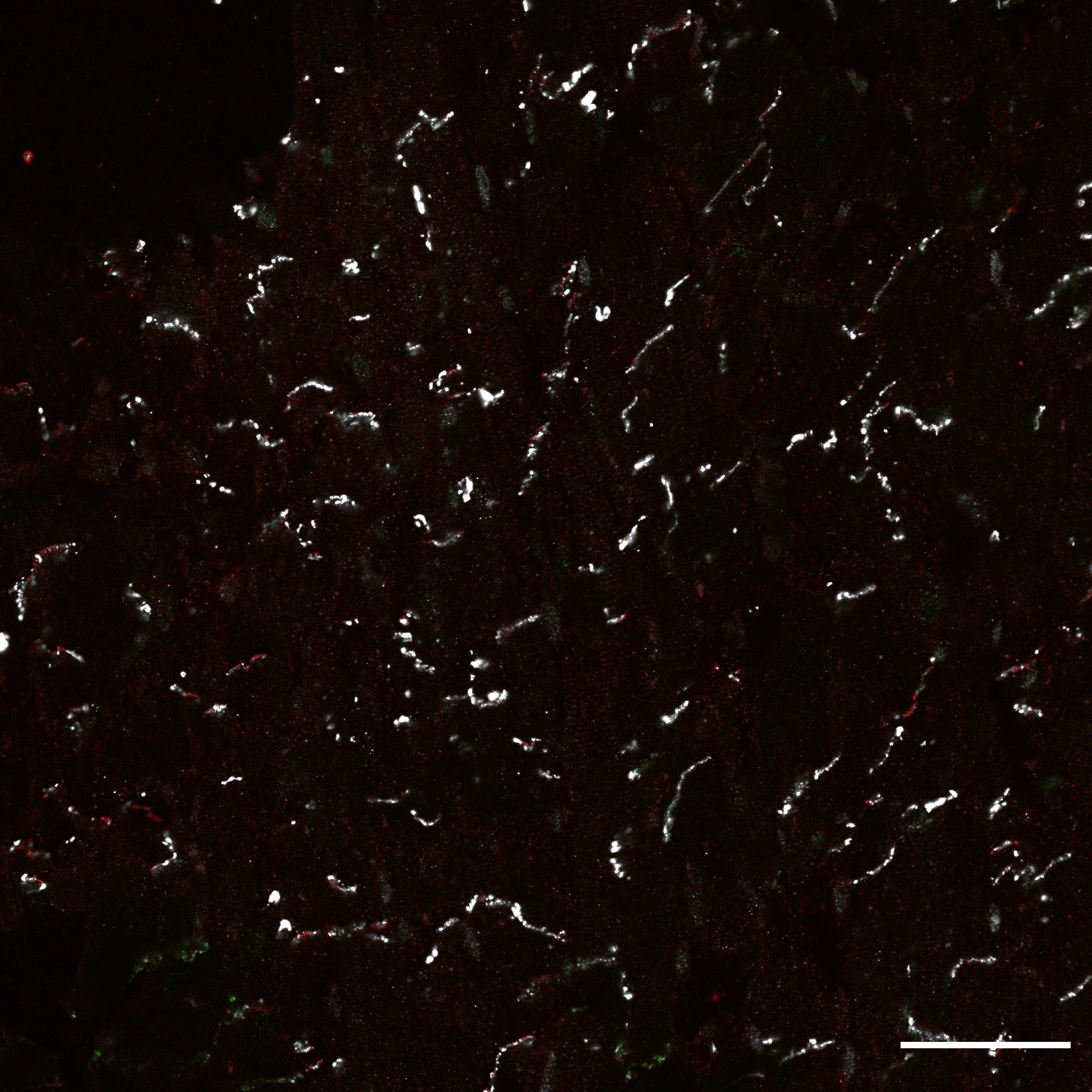

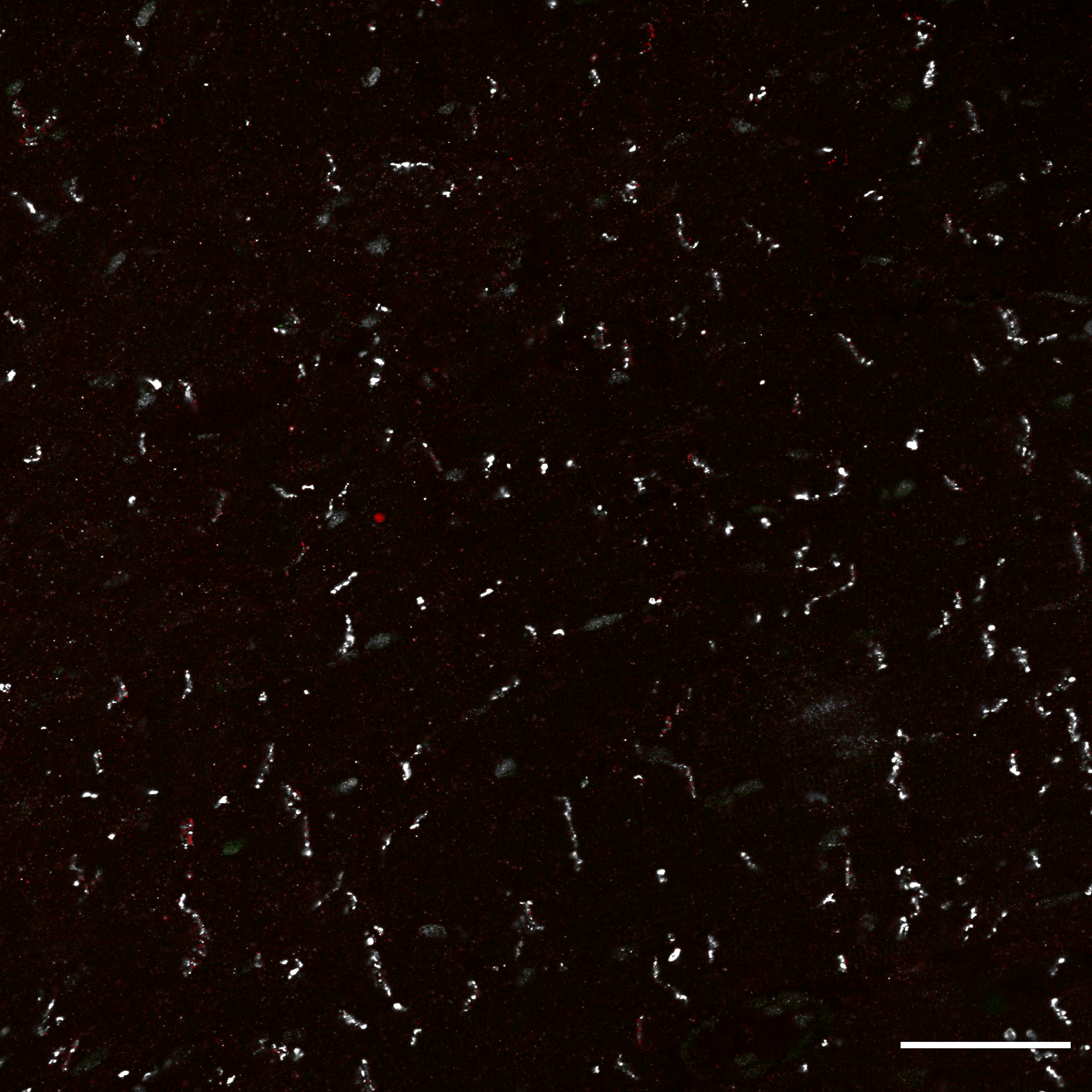

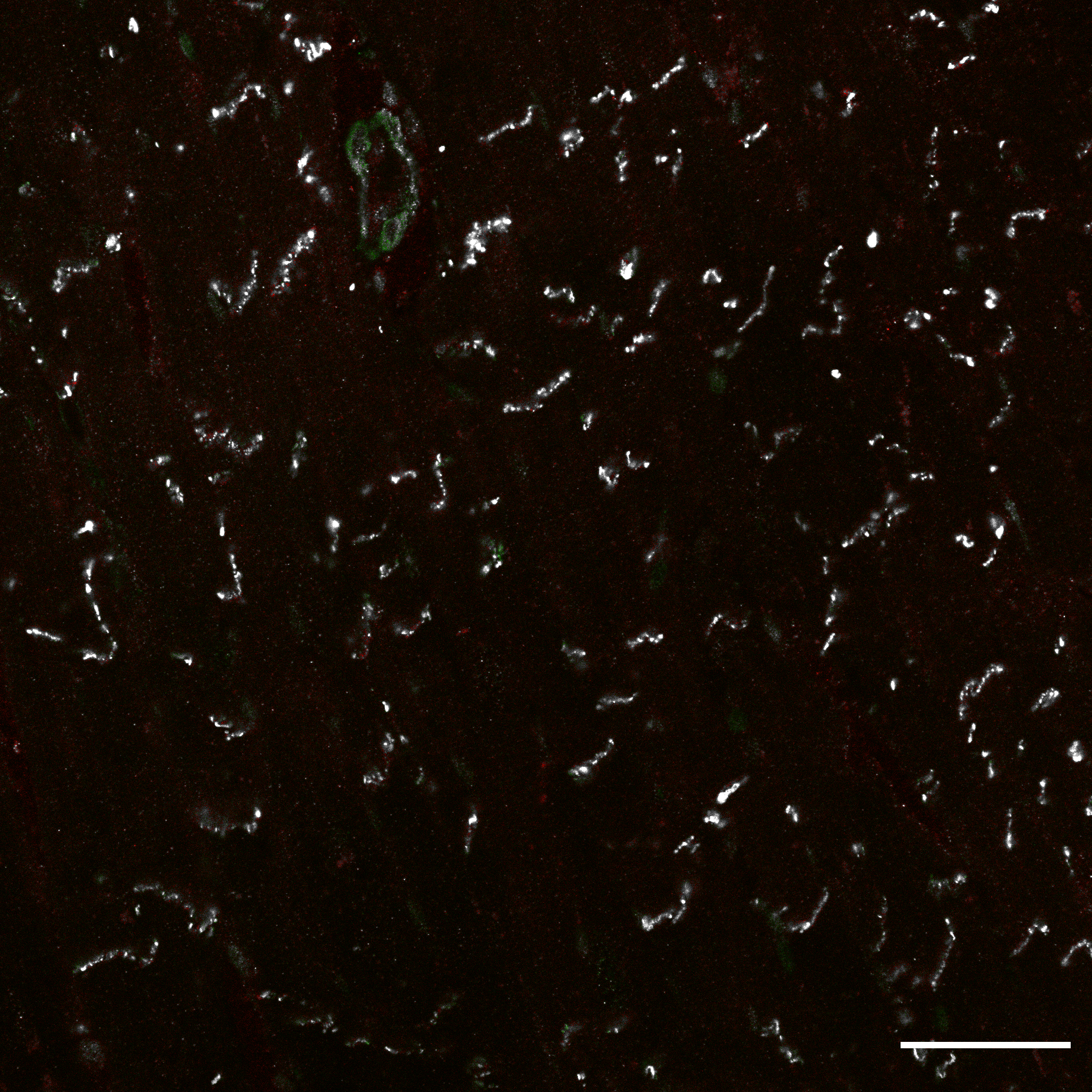


WT


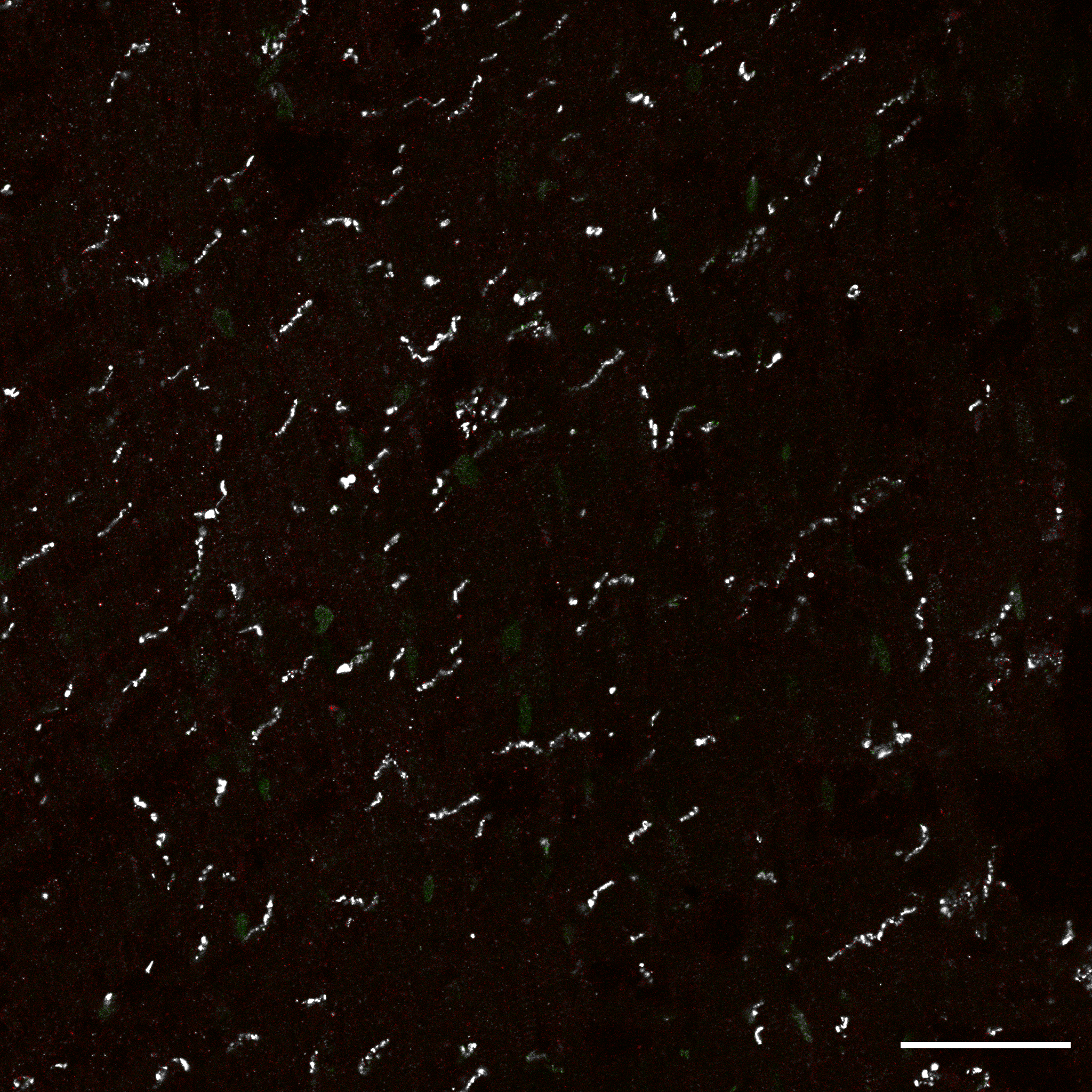

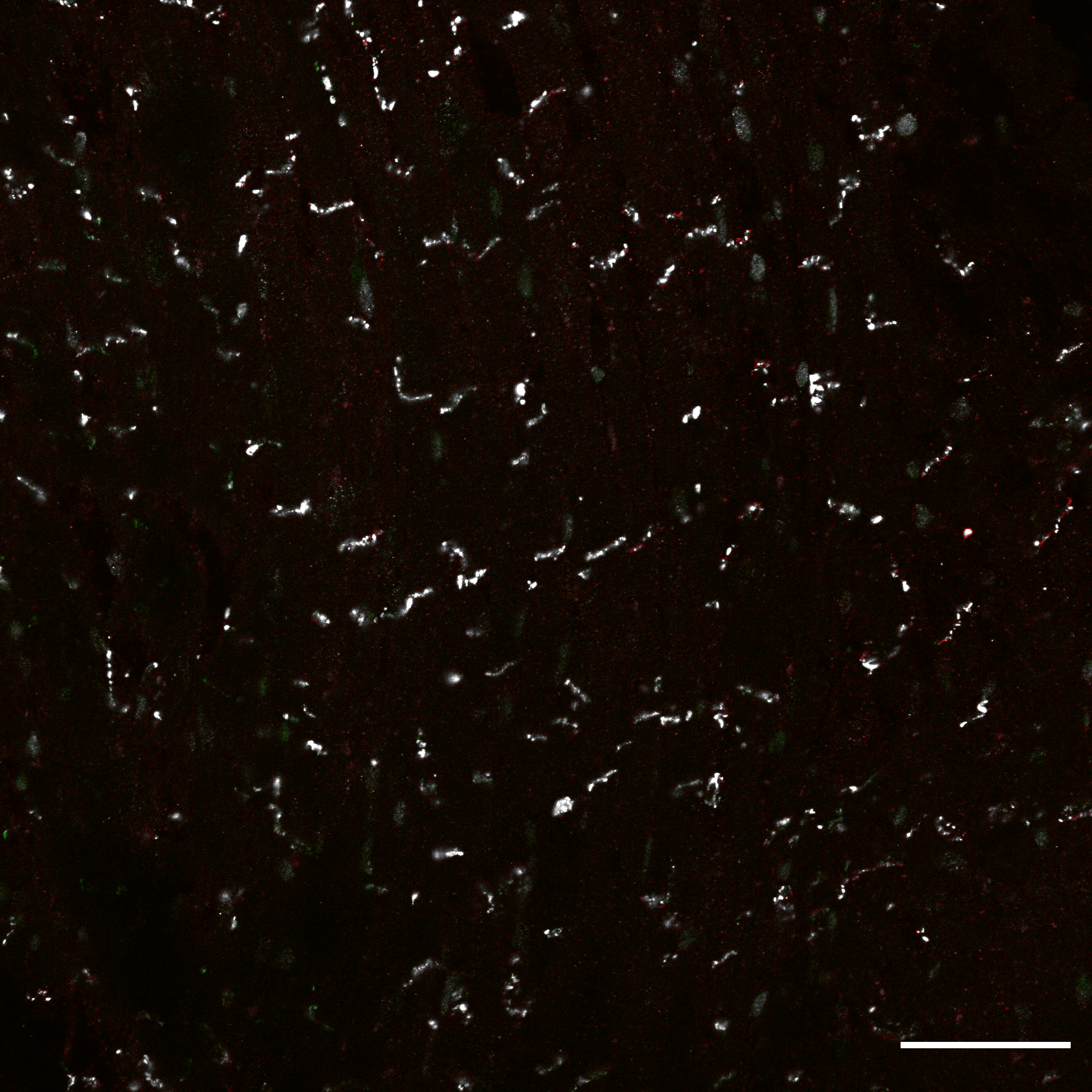

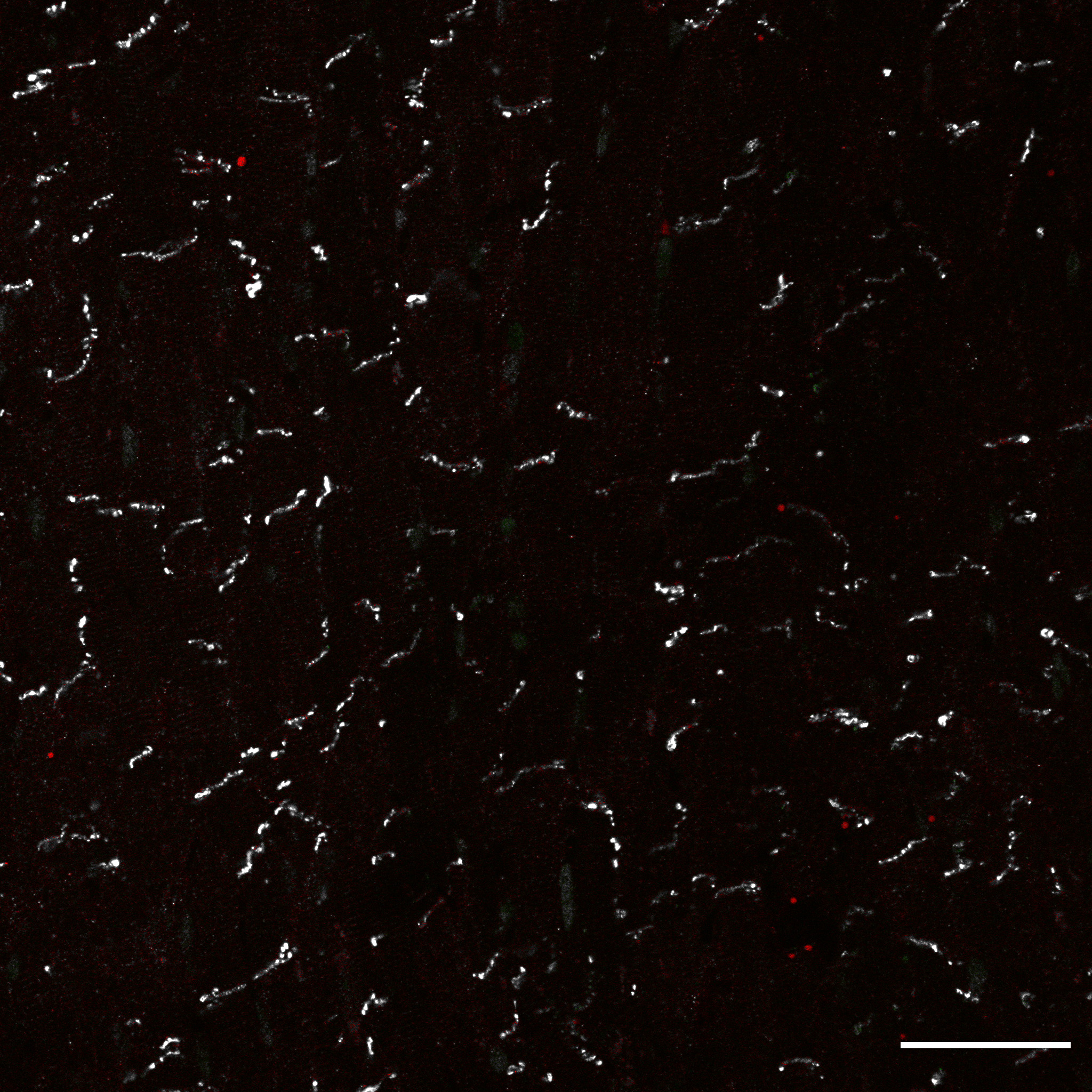


HE

**S3 Fig: Localization of Cx43 at the intercalated disc.** To verify the correct localization of Cx43 at the intercalated disc (ID) we performed immunohistochemistry with N-cadherin as counterstain specific to the IDs. Cx43 was visualized with Alexa 488 while N-cadherin was labeled with Alexa 555. The co-localized pixels from the two channels are depicted as white pixels in the images, exemplarily indicated by white arrows. We found a high grade of co-localized pixels at the ID in both genotypes at all time points, confirming the accumulation of Cx43 at the IDs (scale bar: 50 µm).
