# Supplementary figures and images for: Beneficial effect of voluntary physical exercise in Plakophilin2 transgenic mice
Source: PLoS One. 2021 Jun 4;16(6):e0252649. doi: 10.1371/journal.pone.0252649 (PMC8177441; doi:10.1371/journal.pone.0252649)

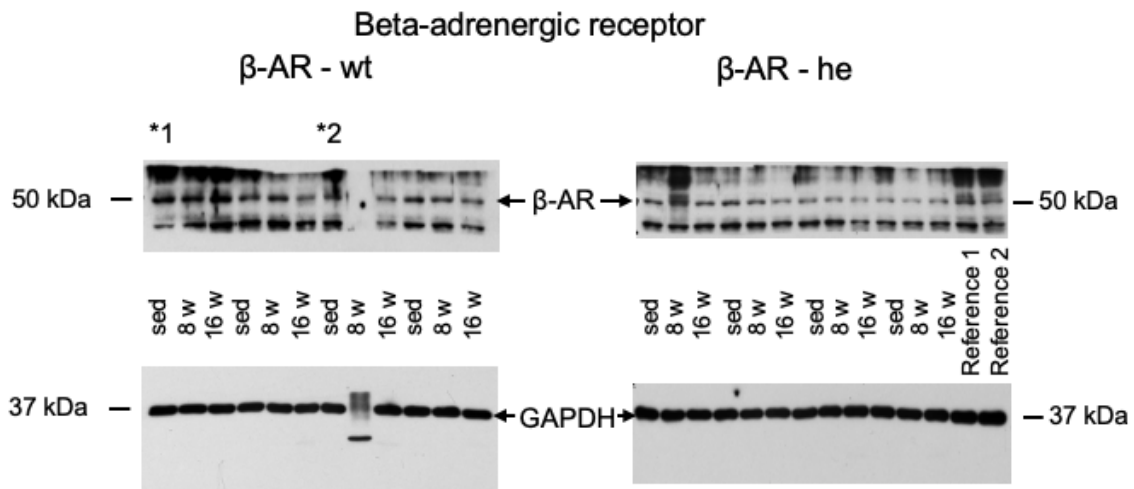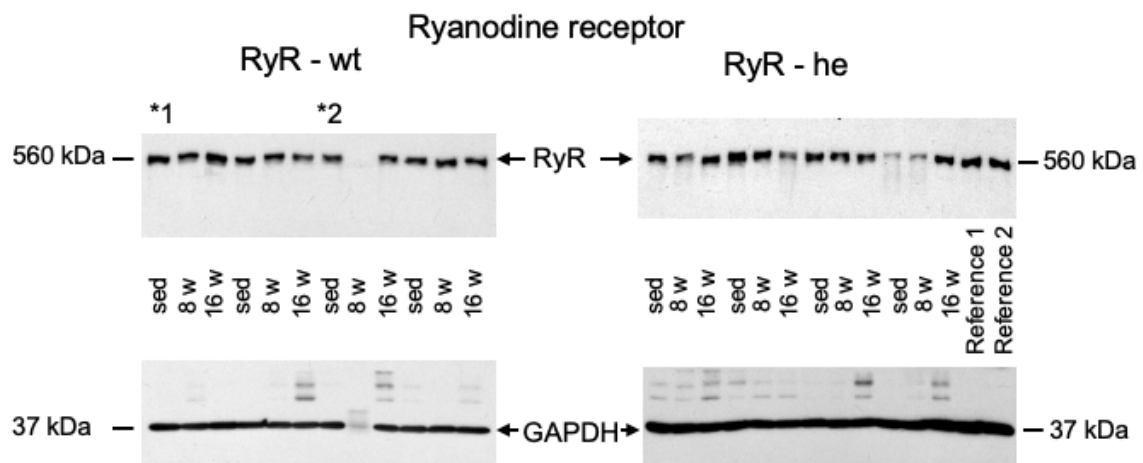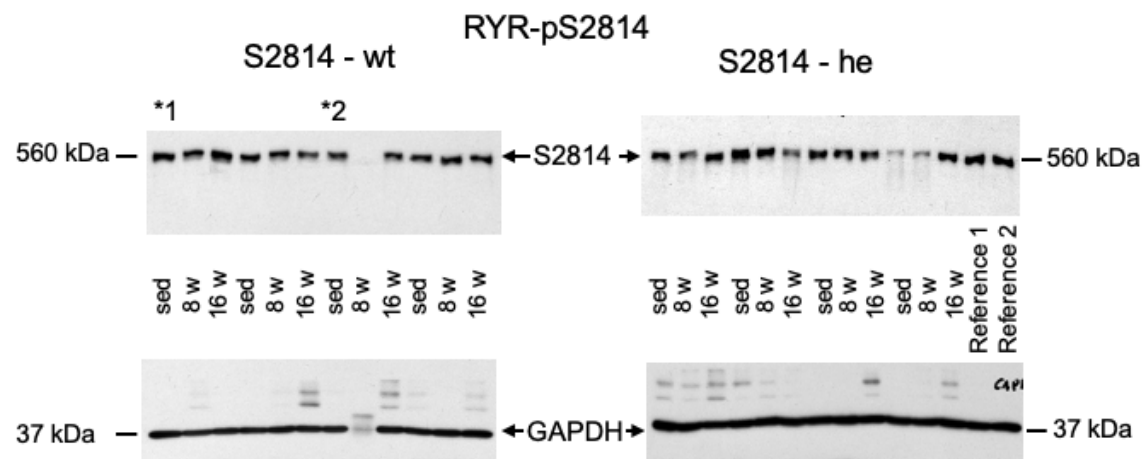

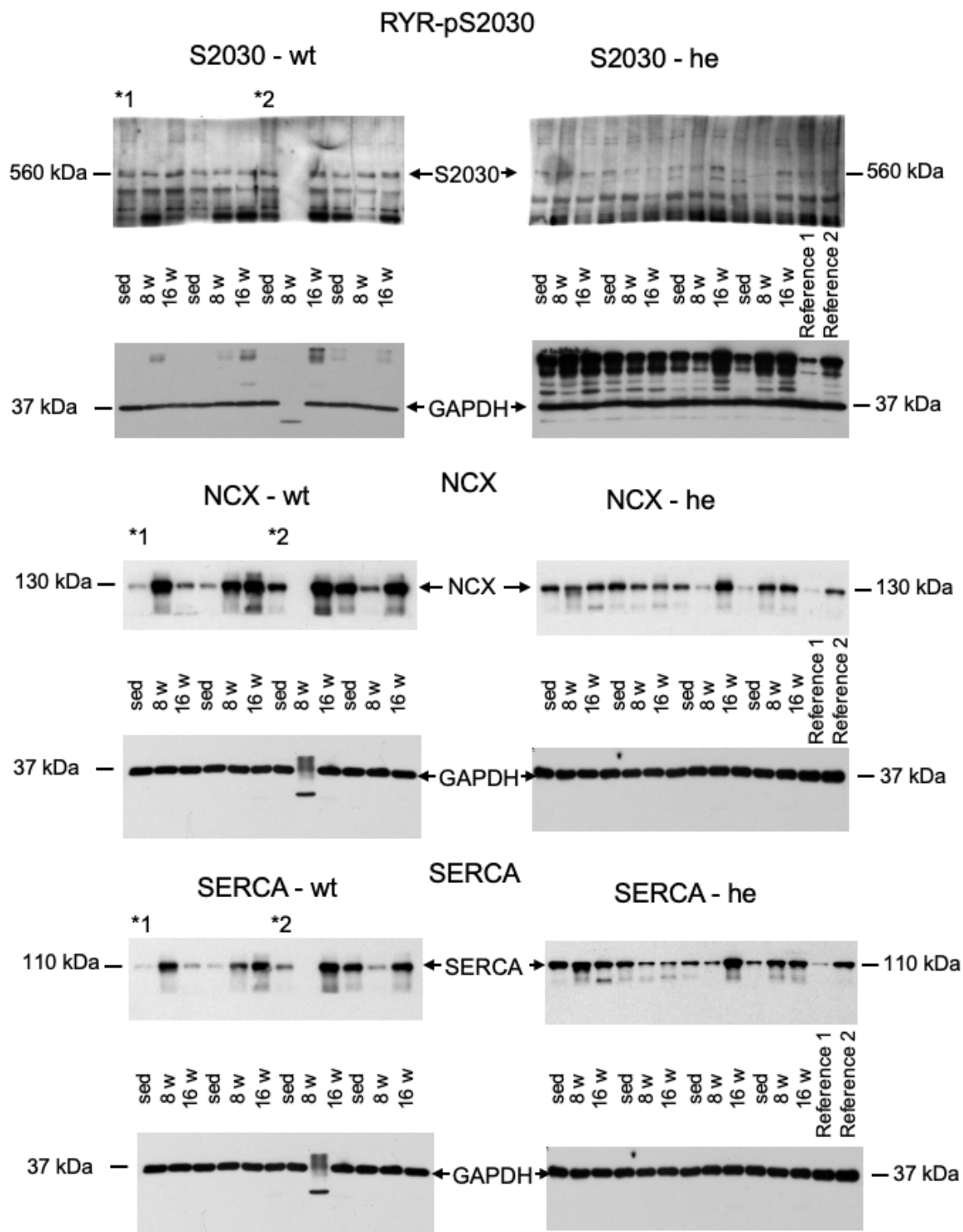

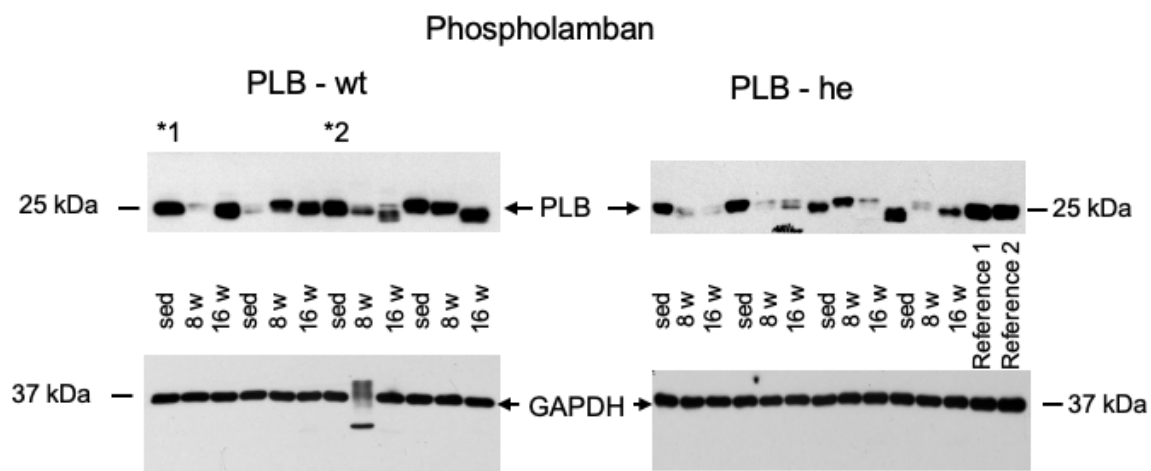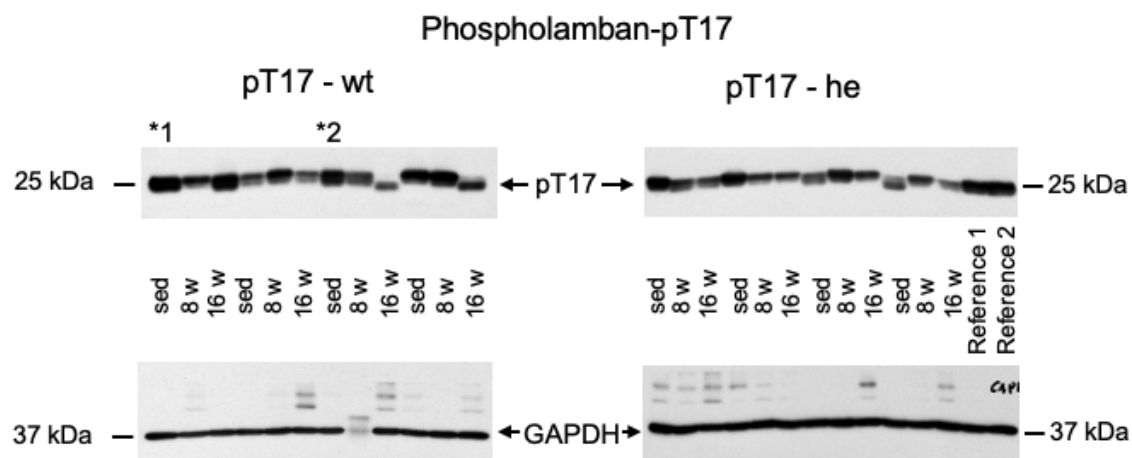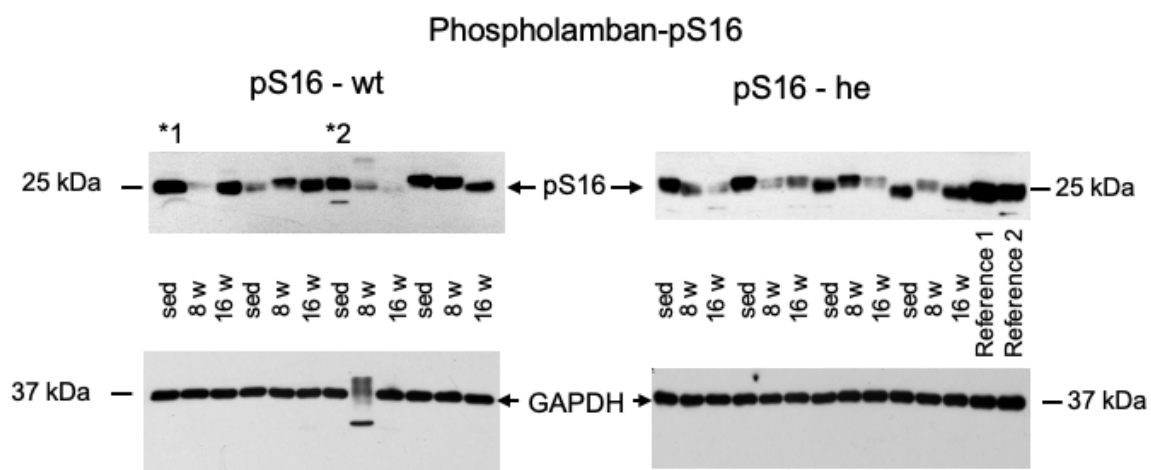

Supplement: S1 Raw images — (PDF) [file pone.0252649.s002.pdf]
